# Light‐Activated Carbon Monoxide Prodrugs Based on Bipyridyl Dicarbonyl Ruthenium(II) Complexes

**DOI:** 10.1002/chem.202002139

**Published:** 2020-08-13

**Authors:** Stepan Geri, Tereza Krunclova, Olga Janouskova, Jiri Panek, Martin Hruby, Daniel Hernández‐Valdés, Benjamin Probst, Roger A. Alberto, Constantin Mamat, Manja Kubeil, Holger Stephan

**Affiliations:** ^1^ Institute of Radiopharmaceutical Cancer Research Helmholtz-Zentrum Dresden-Rossendorf Bautzner Landstrasse 400 01328 Dresden Germany; ^2^ Department of Biological Models Institute of Macromolecular Chemistry Heyrovsky Square 2 16206 Prague Czech Republic; ^3^ Supramolecular Polymer Systems Institute of Macromolecular Chemistry Heyrovsky Square 2 16206 Prague Czech Republic; ^4^ Department of Chemistry University of Zurich Winterthurerstr. 190 8057 Zurich Switzerland

**Keywords:** anti-apoptotic activity, anti-proliferative, cellular localisation, photoCORM, ruthenium(II)

## Abstract

Two photoactivatable dicarbonyl ruthenium(II) complexes based on an amide‐functionalised bipyridine scaffold (4‐position) equipped with an alkyne functionality or a green‐fluorescent BODIPY (boron‐dipyrromethene) dye have been prepared and used to investigate their light‐induced decarbonylation. UV/Vis, FTIR and ^13^C NMR spectroscopies as well as gas chromatography and multivariate curve resolution alternating least‐squares analysis (MCR‐ALS) were used to elucidate the mechanism of the decarbonylation process. Release of the first CO molecule occurs very quickly, while release of the second CO molecule proceeds more slowly. In vitro studies using two cell lines A431 (human squamous carcinoma) and HEK293 (human embryonic kidney cells) have been carried out in order to characterise the anti‐proliferative and anti‐apoptotic activities. The BODIPY‐labelled compound allows for monitoring the cellular uptake, showing fast internalisation kinetics and accumulation at the endoplasmic reticulum and mitochondria.

## Graphical abstract

**Bright and harmful**: A BODIPY dye has been connected to a bipyridyl dicarbonyl ruthenium(II) complex by the Staudinger ligation. The cellular uptake of the complex can be monitored by its green fluorescence. Exposure to UV light induced quick monodecarbonylation accompanied by cell damage.

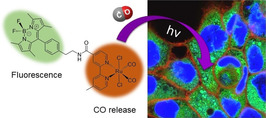

## Introduction

Carbon‐monoxide‐releasing molecules (CORMs) represent a promising prodrug approach since it is known that CO plays a beneficial role in mammals, showing anti‐bacterial, anti‐apoptotic, anti‐proliferative and anti‐inflammatory effects.^1^ To control the administration of the highly toxic gas in a safe manner, certain carbonyl compounds have been designed in which CO is either covalently bound in organic molecules^2^ or coordinated to metal centres1b, 1f, 3 in inorganic complexes. Transition‐metal carbonyl complexes are particularly suitable because they feature a rich chemistry and the M−CO bond strength (π acceptor interaction) can be manipulated, for example, through oxidation or electronic excitation by means of external light irradiation, being triggered by enzymes, ligand exchange reactions, thermal or by pH changes.1b, 1f, 3b, 3c, 4 Several CORMs, especially the widely investigated and prominent example of transition‐metal complexes [Ru(CO)_3_Cl(glycinate)] (CORM‐3) and derivatives of boranocarbonate Na_2_[H_3_BCO_2_] (CO‐A1)^5^ gained considerable attention due to their interesting properties in vitro and in vivo.1b, 1c, 6 However, these classes of CORMs tend to release the CO molecules in a hardly controllable way. For that reason, photoCORMs have been developed inter alia to only liberate the CO upon exposure to light of certain wavelengths, allowing for dosage control in affected tissues/organs as well as liberation at desired locations.3d, 4, 7 To be useful for therapeutic applications, photoCORMs must fulfil various requirements, that is, stability and appropriate solubility under physiological conditions, sufficient high decarbonylation rate constants, non‐toxicity of the “non‐activated” photoCORM and utilisation of biocompatible wavelengths (preferably near‐infrared light for which living tissues are permeable).^8^ To test and proof the therapeutic potential of metal‐based photoCORMs, many anti‐proliferative studies were conducted with rhenium(I)‐ and manganese(I)‐based photoCORMs using UV or blue‐light. Exemplary studies revealed an unspecific toxicity in human cancerous cell lines such as colon adenocarcinoma (HT29), cervical (HeLa), ovarian (A2780), cisplatin‐resistant ovarian (A2780CP70) and breast cancer (MDA‐MB‐231), assuming that the toxicity was induced by the released CO.7h, 9 However, the exact mechanism of pharmacological activity is often unclear and needs further investigation.

**Scheme 1:**
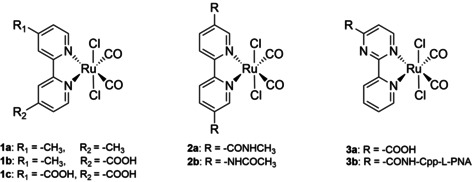
Chemical structures of *trans*‐(Cl),*cis*‐(CO)‐ruthenium(II) dicarbonyl complexes with bipyridyl ligands substituted at 4,4′‐position (**1 a**–**c**) or at 5,5′‐position (**2 a**,**b**) and with pyrimidyl ligands (**3 a**–**c**).

Although Ru^II^ dicarbonyl complexes based on 2,2′‐bipyridine derivatives substituted at 4,4′‐**1** and 5,5′‐positions **2** (Scheme 1) are known as effective CO releasers,^10^ surprisingly, little if any biological studies on this class of Ru‐CORMs are reported so far. These systems are characterised to undergo a stepwise decarbonylation upon UV irradiation with relatively high rate constants.10b, 10c Previously, we reported about the influence of the electron‐withdrawing carboxyl and electron‐donating methyl groups attached to the bipyridyl ligand of Ru^II^ dicarbonyl complexes **1** on the rate of photolysis in organic and aqueous solvent systems. Electronic properties of the ancillary ligands enable a decrease in metal d‐electron density and, thus supports the release of CO. The study clearly revealed a stepwise decarbonylation process with rate constants showing a difference of one order of magnitude between each other and a solvent dependency.10b The second rate constant depends in particular on the electronic structure of the substituent, that is, with coligands bearing electron‐withdrawing‐groups, such as carboxylic groups, the complexes tend to release the CO slower than complexes in which ligands bear electron‐donating groups, such as methyl groups. This is also true when the carboxylic group is replaced by the amide function. Importantly, the CO release is faster when a carboxamide group is connected via the amide nitrogen atom to the bipyridyl skeleton (**2 b**) compared to C‐connection (**2 a**).10d Ru^II^ dicarbonyl complexes based on 2‐(2′‐pyridyl)pyrimidine (cppH) ligands **3** are less efficient CO releasers than bipyridine‐based complexes **1** and **2**. There are no significant differences in CO release properties when the carboxylic (**3 a**) is replaced by an amide group (**3 b**).10a

Neither conjugation of biomolecules or fluorescent dyes nor detailed investigations of the influence of amide functionalization on the CO release properties of bipyridyl dicarbonyl Ru^II^ complexes **1** have been reported so far and biological properties are scarce.

**Scheme 2:**
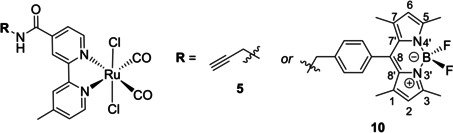
Chemical structures of Ru^II^ dicarbonyl bipyridyl complexes **5** and **10**.

Herein, we report on the preparation and characterisation of two amide‐functionalised (4‐position) ruthenium(II) dicarbonyl bipyridyl complexes bearing an alkyne group (complex **5**) and a fluorescence tag (complex **10**), consisting of a BODIPY (boron‐dipyrromethene) dye, respectively (Scheme 2). BODIPY dyes are a class of organic fluorophores, which exhibit excellent photochemical stability and spectroscopic properties useful for biological systems,^11^ for example, to study the cellular uptake and localisation of the BODIPY‐labelled compounds. As reported recently, conjugates of metal complexes with BODIPY derivatives offer easy access to new theranostics.^12^

We examined the photolysis of these UV‐light‐sensitive Ru^II^ complexes and compare their antiproliferative activities against two human cell lines, epidermoid carcinoma (A431) and embryonic kidney cells (HEK293) with reported structures (Scheme 1). Cellular uptake behaviour and cellular localisation of the fluorescent Ru‐CORM‐BODIPY conjugate **10** using laser scanning confocal microscopy are reported in detail.

## Results and Discussion

### Synthesis of alkyne‐ and BODIPY‐functionalised Ru^II^‐CORM complexes

Starting from 4′‐methyl‐2,2′‐bipyridine‐4‐carboxylic acid (**1 b**), an amide group was introduced to the bipyridine skeleton in different ways. It is essential to prepare the respective ligand prior to the complexation with the ruthenium precursor.

**Scheme 3:**
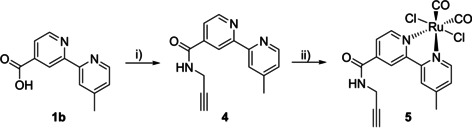
Synthesis of alkyne‐functionalised Ru^II^ dicabonyl bipyridyl complex **5**. Reagents and conditions: i) EDC⋅HCl, HOBt, propargylamine, DIPEA, DMF, r.t., 24 h, yield 61 % of **4**; ii) **4**, [RuCl_2_(CO)_2_]_*n*_, DMF, 90 °C, 30 min, darkness, yield 59 % of **5**.

The alkyne‐functionalised bipyridyl ligand **4** was prepared in 61 % yield from the commercially available carboxylic acid‐containing precursor **1 b**, which was reacted with propargyl amine under peptide coupling conditions with EDC and HOBt (Scheme 3). In the second step, the ruthenium complex **5** was obtained by heating the ruthenium polymer precursor [RuCl_2_(CO)_2_]_*n*_ with **4** under argon in the dark to afford [Ru^II^(Bipy)Cl_2_(CO)_2_] **5** as *trans*‐(Cl) isomer in a yield of 59 %. The *cis*‐(Cl) isomer has not been found by this method.

The ^1^H NMR spectra of **4** and **5** in CD_3_CN as well as ^1^H/^13^C NMR spectra in [D_6_]DMSO and FTIR data of complex **5** are displayed in the Supporting Information (Figures S1–S3). As a result, a distinctive up‐field shift is observed for the proton signals of ruthenium(II) complex **5** compared to ligand **4** in the ^1^H NMR spectrum (Figure S1). Complex **5** shows nine signals. Six resonances are assigned to the aromatic protons of the bipyridine skeleton and three signals to the methyl (*δ*=2.62 ppm), methylene (*δ*=4.19 ppm) and alkyne (*δ*=3.27 ppm) protons. The two characteristic carbonyl ligands are observed in the ^13^C NMR spectrum with chemical shifts of 196.3 and 196.1 ppm in [D_6_]DMSO (Figure S2). Additionally, the vibrations in the IR spectrum at 2073 and 2016 cm^−1^ correspond to the CO ligands (Figure S3). The CH‐ and amid‐alkyne vibrations are observed at around 3300 cm^−1^. The infrared vibration of the CC triple bond is weak and is overlaid with one of the strong CO vibrations.

**Scheme 4:**
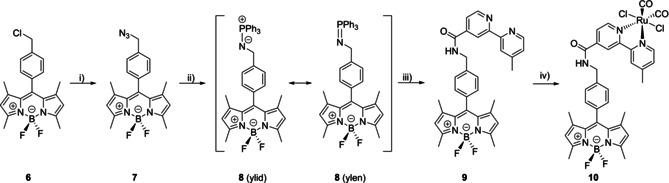
Synthesis of CORM‐BODIPY compound **10**. *Reagents and conditions*: i) NaN_3_, KI, DMF, 45 °C overnight; yield >99 % of **7**; ii) PPh_3_, DMF, r.t., 10 h; iii) **1 b**, DCC, HOBt, DMF, r.t., 16 h; iv) DMF, r.t., 16 h; yield 47 % of **9**; (iv) [RuCl_2_(CO)_2_]_*n*_, MeOH, reflux, 2 h, darkness, yield 68 % of **10**.

An elegant synthetic pathway for the introduction of an amide group via biorthogonal click reaction is the Staudinger ligation, in which an azide reacts with a functionalised triarylphosphine.^13^ A corresponding synthesis approach to reach the green fluorescent BODIPY‐CORM **10** derivative is shown in Scheme 4. At first, the BODIPY moiety was prepared by a standard procedure starting from 2,4‐dimethylpyrrole and 4‐(chloromethyl)benzoyl chloride.^14^ Next, the chloride function at the benzyl position of **6** was substituted by the azide group to yield **7**. Afterwards, the azide‐containing BODIPY derivative **8** was treated with triphenylphosphine to form a resonance‐stabilised iminophosphorane intermediate **8** (not isolated) under elimination of N_2_. Subsequent reaction of **7** with compound **1 b**, which was converted beforehand into the benzotriazolyl ester (not isolated), led to compound **9** in a stepwise Staudinger ligation approach.^15^ The last step involved the complexation of **9** with the Ru‐precursor [RuCl_2_(CO)_2_]_*n*_ under argon and exclusion of light to give complex **10** exclusively as *trans*‐(Cl) isomer in 68 % yield after crystallisation. The formation was confirmed by ^1^H NMR spectroscopy, showing just six expected signals corresponding to bipyridine protons (*δ*=9.76–7.72 ppm) rather than 12 for the *cis*‐(Cl) isomer.10d The characteristic signals of the BODIPY‐core were confirmed using ^1^H, ^11^B, and ^19^F NMR spectroscopy. The methyl and heteroaromatic protons appear at *δ*=1.36, 2.44, and 6.17 ppm. The ^11^B and ^19^F NMR signals are found in the expected region at *δ*=0.6 ppm and *δ*=−143.7 ppm, respectively. The characteristic resonances for the two CO ligands of **10** appear at *δ*=196.3 and 196.1 ppm in the ^13^C NMR spectrum and were evidenced by the symmetric and anti‐symmetric vibrations at 2079 cm^−1^ and 2008 cm^−1^ in the IR spectrum.

### Light‐induced decarbonylation monitored by UV/Vis, FTIR and ^13^C NMR spectroscopy

**Figure 1:**
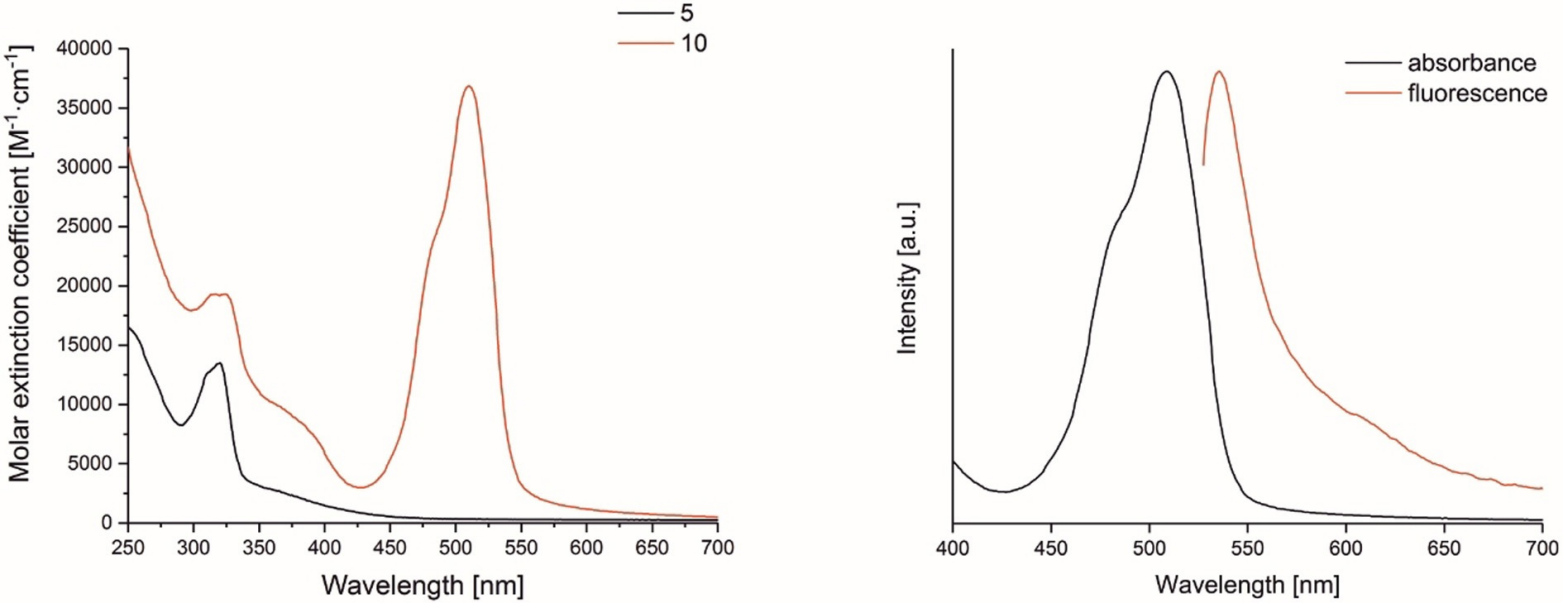
Left: UV/Vis absorption spectra of **5** (50 μm, black line) and **10** (30 μm, red line) in water containing 0.8 % (v/v) DMSO; Right: absorbance (black line) and fluorescence (red line) spectra illustrating the narrow Stokes shift of complex **10**.

UV/Vis absorbance spectra of **5** and **10** in water containing 0.8 % (v/v) DMSO are shown in Figure 1. It must be noted that the complexes are soluble in the respective solvent up to concentrations of 100 μm for **5** and 50 μm for **10**. Extinction coefficients at all absorption maxima as well as at 350 nm were summarised in Table 1. Similar absorption maxima have been reported for Ru^II^ dicarbonyl complexes bearing bipyridyl, pyrimidyl and terpyridyl ligands.10a, 10b, 10c Two absorbance bands at 313 and 324 nm and a shoulder at around 370 nm (Table 1 and Figure 1, left, black line) are observed for the alkyne‐functionalised complex **5**. The transitions above 350 nm are assigned to metal‐to‐ligand and a ligand‐to‐ligand charge‐transfer (MLCT/LLCT) bands. The other two electronic transitions are attributed to π bpy‐to‐π* bpy LLCT bands.10c

**Table 1:** Absorption maxima and molar extinction coefficients of complex **5** and **10** in water containing 0.8 *%* DMSO (*v*/*v*) at room temperature.

Complex	*E*_*λ*max_ [m ^−1^ cm^−1^] (*λ*_max_ [nm])	*E*_*λ*max_ [m ^−1^ cm^−1^] (*λ*_max_ [nm])	*E*_*λ*max_ [m ^−1^ cm^−1^] (*λ*_350_ [nm])	*E*_*λ*max_ [m ^−1^ cm^−1^] (*λ*_max_ [nm])
**5**	12 900 (313)	13 500 (324)	3160 (350)	–
**10**	19 300 (313)	19 300 (324)	11 100 (350)	36 800 (509)

As found for **5**, the Ru^II^‐CORM‐BODIPY complex **10** exhibited the same absorption maxima in the UV range at 313, 324 nm and a more significant shoulder at approximately 370 nm. A new band in the visible region at 509 nm appeared in the absorbance spectrum (Figure 1, left, red line). The transition band at 509 nm is characteristic for the BODIPY core and it is due to π BODIPY–π* BODIPY transitions.^16^

An intense emission band was observed at 539 nm (*λ*_ex_=509 nm), which corresponds to a relatively large stokes shift (Figure 1, right). Typically, narrow stokes shifts and intensive fluorescence are characteristic features of BODIPY derivatives.11a

To evaluate the photolabilities of both compounds **5** and **10**, photolysis experiments were carried out upon light exposure at 350 nm. In general, the photo‐induced decarbonylation of the complexes [RuCl_2_(L)(CO)_2_] (L=bpy and its derivatives) causes the cleavage of the CO ligands from the coordination sphere when external light of high energy (UV range) is applied. The vacant site is replaced by solvent molecules. The mechanism has been previously elucidated by our group using multivariate curve resolution alternating least‐squares (MCR‐ALS) as well as by other groups with time‐resolved IR spectroscopy and DFT calculations.10b, 10c, 17 Usually, the release of the first CO occurs very rapidly (pico‐second range),10c while the second CO proceeds substantially slower. In fact, we have reported that decarbonylation is solvent‐dependent and the rate constants in aqueous systems were an order of magnitude slower than in organic solvents. However, the first rate constant is still high, accompanying with a very quick release of the first CO ligand. Interestingly, the rate constants in aqueous systems were less affected by electronic influences of ancillary ligands than in acetonitrile.10b

**Figure 2:**
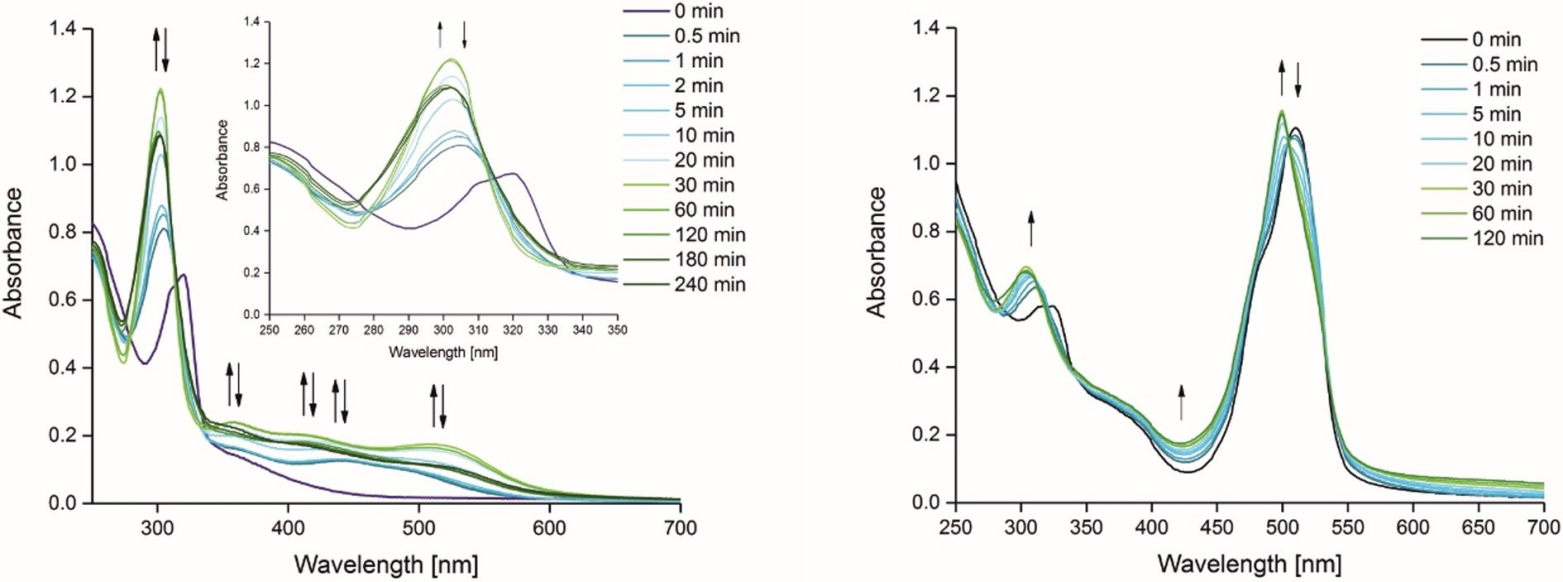
UV/Vis absorption spectra measured for **5** (50 μm, left) and **10** (30 μm, right) in water containing 0.8 % (*v*/*v*) DMSO after different periods of exposure to 350 nm of radiation (*E*_v_≈6 mW cm^−2^) at room temperature.

Changes in the absorbance of **5** and **10** in water containing 0.8 % (*v*/*v*) DMSO upon irradiation at 350 nm were monitored over time by UV/Vis spectroscopy (Figure 2). Within the first seconds of exposure to 350 nm (*E*_v_≈6 mW cm^−2^), the spectra changed substantially for **5**. The bands at 313 and 324 nm diminished and a new band at 304 nm grows in. The shoulder at around 370 nm shifted bathochromically and new broad bands appear between 400 and 550 nm. All these bands reached a maximum extinction within 30 min, but decreased after 1 h of irradiation. A similar, time‐dependent spectral behaviour is apparent for ruthenium(II) dicarbonyl compounds bearing similar bipyridyl ligands.10b

Complex **10** behaves comparably since it exhibits similar electronic excited states in the UV range. The former bands at 313 and 324 nm became weaker immediately and a new band at 303 nm appeared. The shoulder at about 370 nm is slightly shifted to around 360 nm and increased over time. The π BODIPY–π* BODIPY transition at 509 nm underwent a 10 nm hypsochromic shift and increased in intensity.

**Scheme 5:**
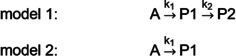
Serial mechanisms (model 1 and 2) for the photoreaction of complex **5** and 10. A=starting complex; P1, P2=photoproducts.

To solve the mechanism and kinetics of the photo‐decarbonylation process, the UV/Vis spectra were analysed by MCR‐ALS, as described previously.10b A kinetic model with three consecutive steps and two individual compounds (A, P1 and P2, Scheme 5) was calculated using the data at 350 nm for complex **5** in water containing 0.8 % (*v*/*v*) DMSO whereas for complex **10** two kinetic models were fitted; model 1 three species with two rate constants and model 2) two species with one rate constant.

The fitted spectra as well as the concentration profiles of **5** and **10** and their photoproducts are shown in Figures S16–S17. For complex **5**, only two spectra were available for the fit, because the first absorbance spectrum drastically changes upon exposure to 350 nm. The rate constant *k*_1_ is considered as a lower limit with *k*_1_>4 min^−1^ and *k*_2_=0.176±0.05 min^−1^. As mentioned above, we determined two kinetic models for complex **10**. Model 1 determined three consecutive steps with two individual components (*k*_1_=1.913±0.067 min^−1^ and *k*_2_=0.063±0.001 min^−1^). Changes were less pronounced for complex **10** than for **5**, and we obtained a *k_1_*≈2 min^−1^, a similar order of magnitude as for **5**. It is thus evident for **5** and **10** that the first rate constants *k*_1_ are around one to two orders of magnitude larger than *k*_2_ confirming a fast first step, which is in agreement with previously published rate constants.10b Since rate constants of **5** are in the same order as for **10**, the kinetics of CO release is little influenced by replacing the carboxylic acid group with a carboxamide group. It should be noted that the concept of kinetic order for photochemical reactions may be misleading. The rate constants discussed here are considered conditional rate constants for the observed exponential decay. However, for comparable measurement conditions and similar extinction coefficients, the determined values of *k_n_* are a good representation of the quantum yield.7c, 18 Moreover, the quantum yield can be directly derived when the photon flux is known (see Experimental Section).

The photo‐induced loss of the CO ligand from the coordination sphere was further investigated by IR and ^13^C NMR spectroscopy. A monodecarbonylation was confirmed by IR and ^13^C NMR spectroscopy for **5** and **10** after 4 h of irradiation (Figures S14 and S15). The two characteristic carbonyl resonances of **5** and **10** of around 196 ppm disappear after irradiation, but several ^13^CO signals between *δ*=200–205 ppm appeared, indicating the formation of several ruthenium(II) monocarbonyl isomers of the general formula [Ru^II^(bpy)Cl_2_(CO)(solvent)]. In addition, the IR spectra confirmed the appearance of the monocarbonyl species after exposure to UV light, showing a single carbonyl vibration band at 1982 cm^−1^ for both Ru^II^ complexes.

**Figure 3:**
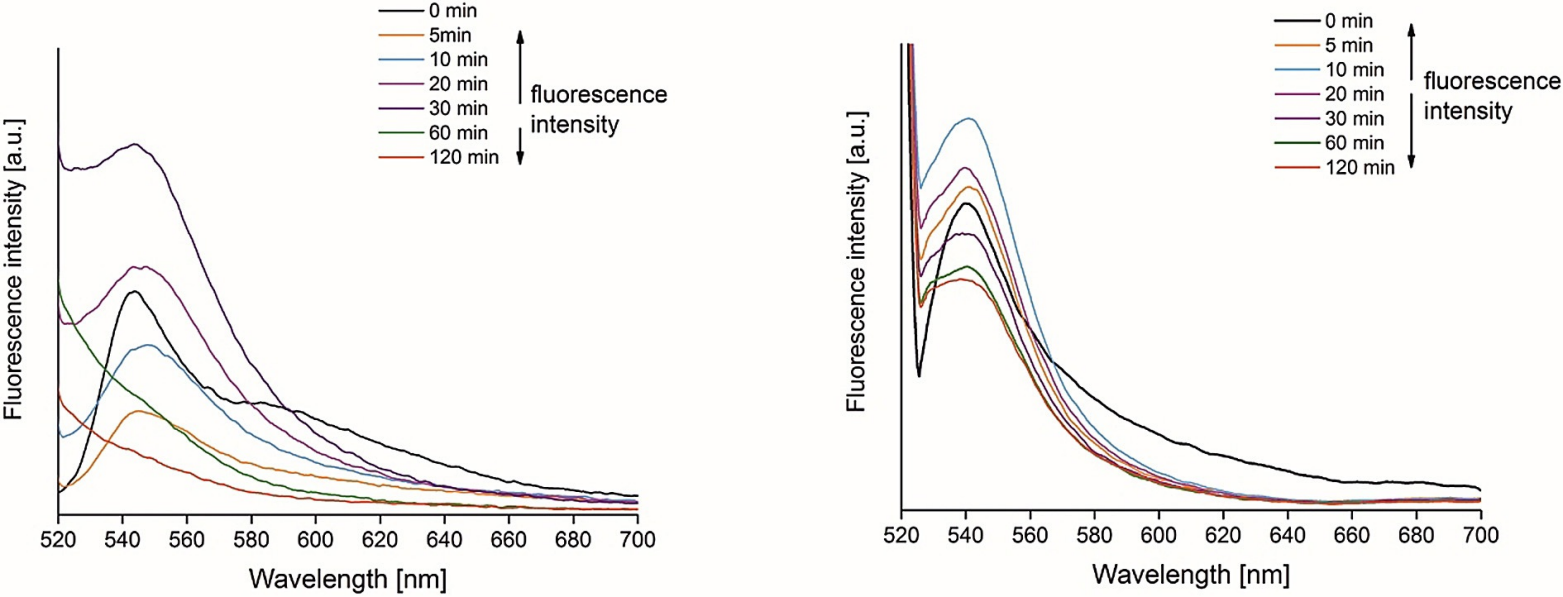
Fluorescence spectra of **9** (left, 30 μm in water containing 0.8 % (*v*/*v*) DMSO) and **10** (right, 30 μm in water containing 0.8 % (*v*/*v*) DMSO) before and after different periods of exposure to 350 nm of radiation (*E*_v_≈6 mW cm^−2^) at room temperature.

The emission spectra of **9** and **10** before and after irradiation for 120 min to 350 nm light are shown in Figure 3. It is worth noting that for compound **10** an increase of fluorescence over time was observed when exposed to UV light, before subsequently the fluorescence intensity started to decrease, indicating the decomposition. However, the control experiment with compound **9** showed complete photobleaching after 60 min of irradiation at 350 nm, suggesting that the metal centre is slowing down the photochemical decomposition process. An UV‐induced fluorescence enhancement has been reported in the presence of a photosensitizer and oxygen molecules due to the production of reactive oxygen species (ROS).^19^

In order to corroborate decarbonylation as the main CO release process, time‐dependent gas chromatography with a thermal conductivity detector (GC‐TCD) was performed. When complex **5** was dissolved in DMSO/water (3:1, *v*/*v*) and was irradiated at 390 nm (0.35±0.02 μE s^−1^) under N_2_, the amount of released CO increased significantly to about 1 equiv. after 4 h (Figure S18). After 20 h, the amount of CO reached almost 1.5 equiv. per Ru, corresponding to 75 % of the theoretical amount of 2 equiv. carbon dioxide was also detected, but the amount of CO_2_ was just slightly increasing within time and retained almost constant at the end. In total, a minor CO_2_ amount of 0.15 equiv. was detected. The oxidation of CO to CO_2_ in the presence of a catalyst in aqueous systems, known as “water gas shift reaction”, can occur.^20^ This reaction usually needs high temperature even in the presence of a catalyst.

### Proliferation studies

The effect of CO is highly cell‐specific and is based on mechanisms that are not yet fully clear. According to the current knowledge, CO exerts a decisive influence on mitochondrial activity by inhibiting the function of cytochrome‐*c*‐oxidase (COX, complex IV), an important enzyme in the respiratory chain of the cell. The disruption of electron transport and the associated oxidative phosphorylation leads to an overproduction of reactive oxygen species. ROS causes single or double strand breaks in the DNA and is ultimately the reason for apoptosis.6a, 21

**Figure 4:**
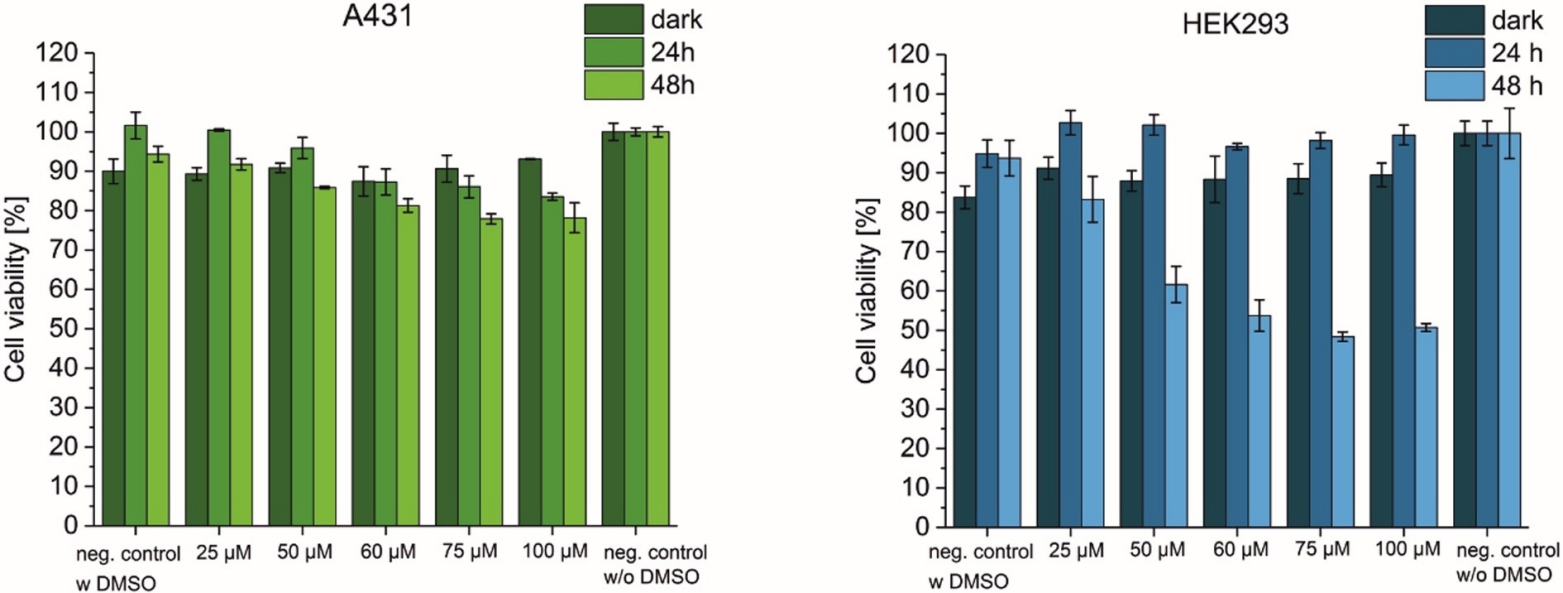
MTS assay of **5** using different concentrations (25, 50, 60, 75 and 100 μm) in A431 (left) and HEK293 (right) cell lines measured after 24 and 48 h after 10 min exposure to 350 nm (*E*_v_≈6 mW cm^−2^) at 37 °C. The percentage of cell viability is expressed relative to untreated cells (ANOVA at *α*=0.05).

**Figure 5:**
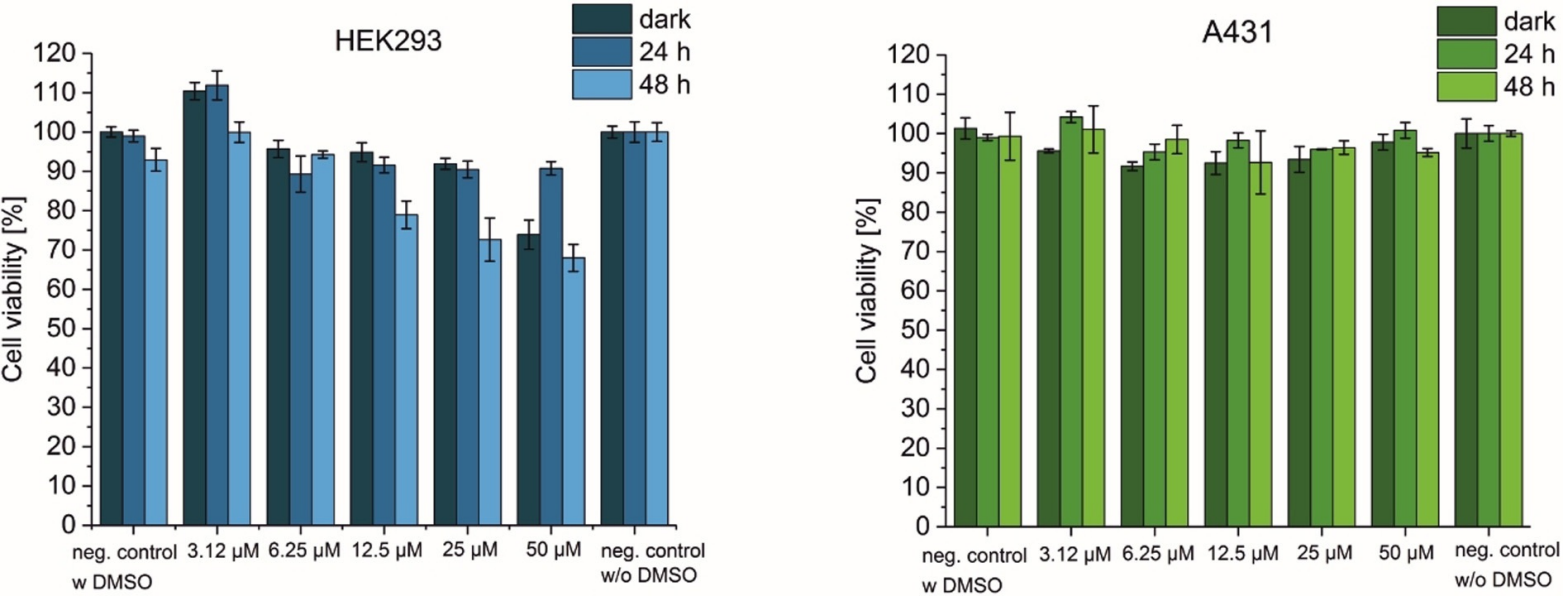
MTS assay of **10** using different concentrations (3.15, 6.25, 12.5, 25 and 50 μm) in A431 (left) and HEK293 (right) cell line measured after 24 and 48 h after 10 min exposure to 350 nm (*E*_v_≈6 mW cm^−2^) at 37 °C. The percentage of cell viability is expressed relative to untreated cells (ANOVA at *α*=0.05).

The pharmacological potential of the synthesised photoCORMs **5** and **10** were investigated in two different human cell lines: epidermoid carcinoma cell line (A431) and a human an embryonic kidney cell line (HEK293) transformed by an adenovirus type 5 (AD5) DNA without (here referred as dark) and with exposure to 350 nm light (*E*_v_≈6 mW cm^−2^) for 10 min at 37 °C. The compounds were dissolved in water/DMSO 0.8 % (*v*/*v*) at concentrations from 3 to 100 μm. The cells were treated with the respective compounds 24 h before exposure to UV light. After 24 h of treatment, the cells were irradiated for 10 min at 350 nm and stored in the incubator for 24 and 48 h prior to cell viability determination by using MTS assay. Untreated cells (without addition of compounds **5** and **10**) with and without DMSO in water as well as kept in the dark and irradiated for 10 min at 350 nm were used as controls. The results are shown in Figures 4 and 5.

The induction of a toxic effect of CO was investigated in both cancerous (A431) and non‐cancerous (HEK293) cells. No differences in cell viability was observed when A431 cells were treated with **5**, while exposed to UV‐A light for 10 min at 37 °C. All treated cells remain viable over the full concentration range.

In contrast, the HEK293 viability decreased significantly after 48 h of exposure to 350 nm light for 10 min while the non‐irradiated cells remained viable. Concentrations above 60 μm reduce the cell viability of irradiated cells by more than 50 %. This observation points out that the non‐cancerous cell line is more sensitive to CO, which induces a toxic effect not observed in the cancerous A431 cell. The results for the fluorescent complex **10** are comparable (incubated for 24 h in the dark in respective cell line, then irradiated at 350 nm for 10 min at 37 °C with subsequently cell viability determination after 24 and 48 h exposure to UV light) in the respective cell lines (Figure 5). Here too, a decrease of HEK293 cell viability is evident already at 12.5 μm after 48 h. At this time, just 68 % of viable cells were obtained, which showed indeed a significant toxicity compared to the controls kept in the dark. In contrast to HEK293 cells, the cancerous A431 cell line seems to be unaffected by the treatment of photoCORM **10**. No significant decrease in cell viability was observed in the concentration range studied neither at 24 nor at 48 h after exposure to UV light.

The results obtained with Ru^II^ compounds **5** and **10** seem contradictory to published proliferation/cytotoxicity studies where anti‐proliferative and apoptotic activities have been described for Mn^I^‐ or Re^I^‐based photoCORMs in various cancerous cell lines.7h, 9a, 9c, 9j It is worth nothing that CO applied as a gas, can act paradoxically as inhibitor or mediator for tumour cell growth.^22^ Ru^II^‐based photoCORMs may undergo a different mechanism of action upon CO release than Mn^I^‐ and Re^I^‐based photoCORMs.

Overall, the results are not easy to interpret, because on the one hand cell lines investigated have different metabolism and on the other the compounds contain several elements, which can have a different influence on proliferation activity. Concerning the latter, there are a number of biological studies with BODIPY derivatives of metal complexes, which indicate that the metal itself and the corresponding complexing agent are primarily responsible for their biological activity.12d For example, the anti‐proliferative activity of platinum complexes is reduced when BODIPY is incorporated.^23^

### Cellular uptake behaviour

BODIPY fluorophores have been used for staining cell compartments for a long time. In this context, fluorescent ceramide derivatives accumulate at the Golgi apparatus and endoplasmic reticulum in particular.^24^ Metal complexes with appended BODIPY moieties can also overcome the outer cell membrane and accumulate in the cytosol/cytoplasm.12a Platinum and organometallic iridium complexes with BODIPY tags are characterised by mitochondrial targeting.^23^, ^25^ Cellular uptake is fast and can be accelerated by the presence of BODIPY.12a A cyclopalladated BODIPY probe (COP‐1) was used for monitoring the CO release in living cells (HEK293T).^26^ In this way, it could be shown that [Ru(CO)_3_Cl(glycinate)] (CORM‐3) was efficiently taken up by the cells and CO was released. Very recently it has been shown that a relatively simple modification of the BODIPY framework leads to organelle‐specific targeting.^27^ However, the processes that determine the targeting are not well understood.

**Figure 6:**
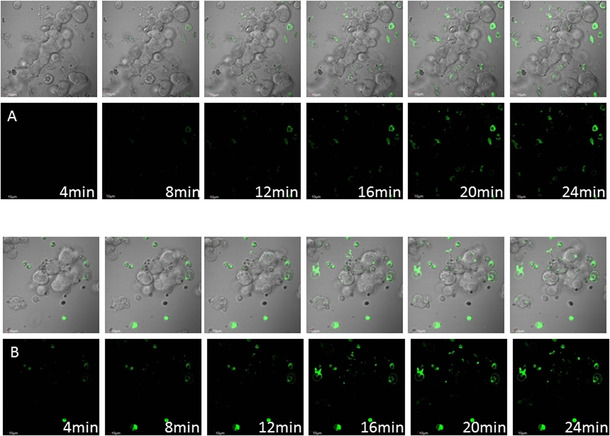
Time monitoring of cellular uptake of compound **10** (10 μm) by A) A431 cells and B) HEK293 cells.

Prior to the irradiation experiments, we monitored the time‐dependent uptake behaviour in A431 and HEK293 cells with the green fluorescent compound **10**. Without signal quantification, it is nicely visible how the penetration of **10** into the cells (Figure 6) within the first few minutes is observed. The uptake is slightly faster in HEK293 cells most likely caused by a different metabolisms. The intracellular localization is similar in mitochondria and endoplasmic reticulum (ER) after 30 min and 4 h as was indicated by Pearson correlation coefficient (PCC),^28^ which is used to describe the colocalisation of fluorescent signals in biological microscopy.

### Colocalisation

**Figure 7:**
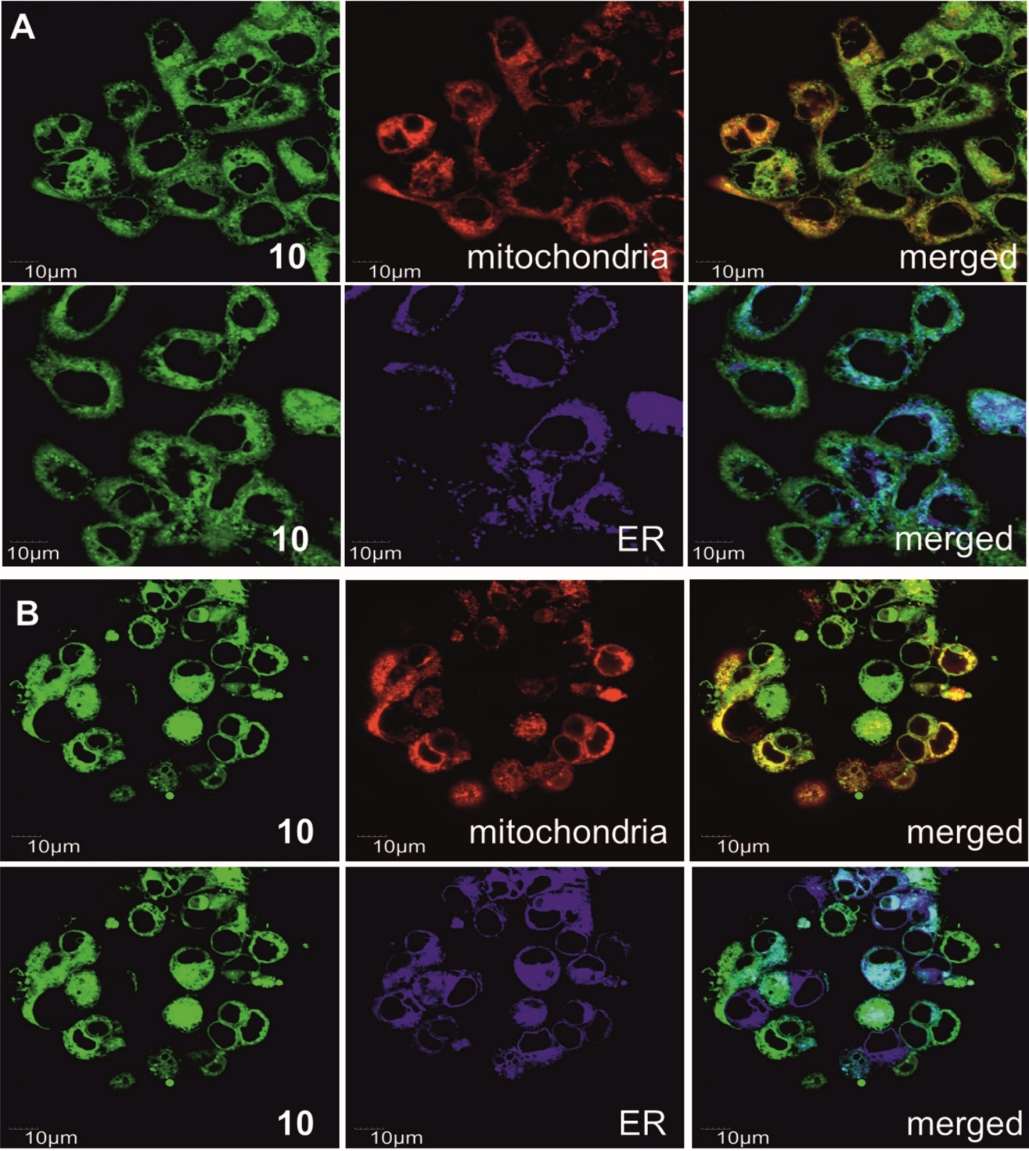
Cellular colocalisation of compound **10** (10 μm) incubated in A) A431 and B) HEK293 cells for 30 min in culture medium with ER and mitochondria after 30 min incubation in A) A431 and B) HEK293 cells. Fluorescence image of **10** (left). Counter stains were then added for 15 min at 37 °C with 5 % CO_2._ Mitotracker^TM^ (0.25 μm) for mitochondrial staining (mitochondria, merged with compound **10** in bottom right) and ER‐Tracker™ Blue‐White DPX (0.5 μm) for endoplasmic reticulum staining (ER, merged with **10** in bottom right). Scale bars are 10 μm.

Fluorescence imaging of A431 and HEK293 cells visualises the intracellular uptake of the green fluorescent BODIPY‐CORM compound **10** after 30 min (10 μm) (Figure 7) and 4 h (50 μm) (Figures S19, S20) of treatment by laser scanning confocal microscopy (LSCM). To analyse the distribution and the localisation in specific cell compartments, the cells were labelled with the counter stains MitoTracker® for mitochondria, ER‐Tracker™ Blue‐White DPX for endoplasmic reticulum, Hoechst33342 for cell nucleus and Cellmask™Deep Red for plasma membrane between 15 and 30 min at 37 °C. As already discussed, compound **10** is taken up rapidly by both cell lines and is spread through the cytoplasm. As expected, an internalisation by the living cells with an unspecific accumulation in mitochondria and endoplasmic reticulum (ER) (Table 2) was detected. The mitochondria are an important target for CORMs. The close proximity to the respiratory chain should enhance the therapeutic outcome, once CO is released. The PCCs are close to one for both investigated organelles (Table 2), which corresponds to a high level of colocalisation. To summarise, no differences in localisation between the different cell lines nor a dependency on incubation time were determined. Moreover, the nuclei membrane are invaginated and the circular shape of nuclei are changed (marked with arrows). However, compound **10** did not penetrate the cell nuclei. As a result, no uptake in the cell nucleus was detected and the shape of deformation is not accompanied with increased toxicity. Similar results were reported elsewhere.^29^ The colocalisation studies revealed similar Pearson correlation coefficients (PCC) quantified in mitochondria and endoplasmic reticulum (ER) after 30 min and after 4 h (Table 2). The Pearson correlation coefficient quantifies the degree of colocalisation in the region of interest. So, for example the PCC correlates the intensities gained from red pixels (mitochondria stained by MitoTracker® staining) and green pixels (compound **10**). Due to the similar PCCs, obtained at different time points, irradiation with UV light can be done already after 30 min of treatment with complex **10**.

**Table 2:** Pearson correlation coefficient of compound **10** determined in mitochondria and ER after 0.5 and 4 h in A431 and HEK293 cell line.

Region of interest	Pearson correlation coefficient			
	A431		HEK293	
	0.5 h	4 h	0.5 h	4 h
mitochondria	0.79	0.86	0.81	0.88
ER	0.77	0.78	0.65	0.9

### Apoptosis and necrosis assay

**Figure 8:**
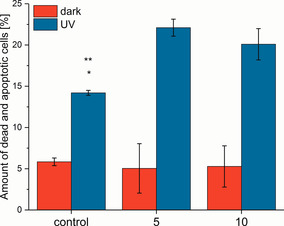
Diagram showing the percentage (*P>0.05, **P>0.01, using Dunnett post test as part of one‐way ANOVA analysis) of dead and apoptotic A431 cells treated without (control containing 0.1 % DMSO) or with complexes **5** and **10** (10 μm containing 0.1 % DMSO) in the dark and after irradiation of UV light (350 nm, UV hand lamp, 6 W) for 10 min. The cells were incubated after irradiation 4 h before analysis.

A431 cells were incubated with compound **5** and **10** for 0.5 h and then irradiated for 10 min with UV light (UV hand lamp, 6W). As controls, we used A431 cells incubated with compound **5** and **10** for 0.5 h, but without irradiation as well as untreated cells which were kept in the dark and irradiated. The percentage of apoptotic and dead cells were evaluated using flow cytometry (Figure 8 and Figure S21 A). Without irradiation, we did not find significant differences between the control (untreated cells) and cells treated with both compounds **5** and **10**. We detected a slightly higher percentage of dead cells (Q1) in the control, which can be caused by the preparation of the sample. However, very few cells were detected, which were in an early or late stage of apoptosis. In comparison, there is a visible shift of the cell population towards the apoptosis sector (Figure S21, compartment Q2 late apoptosis and Q3 early apoptosis) of cells treated with compound **5** and **10**. However, these changes were not significant.

After UV irradiation, we determined an increase of dead and apoptotic cells (Q1+Q2+Q3) for the control (6 % kept in the dark vs. 13 % irradiated, Figure 8), showing the cytotoxic effect of UV radiation. In comparison to the cells treated with both photoCORMs, the percentage of dead and apoptotic cells rose to about 20 % (22.1 % for compound **5**; 20.1 % for compound **10**). These results are significant (*P*>0.1 control vs. **5**; *P*>0.5 control vs. **10**) and thus we can assume that the enhanced cytotoxicity is not only a consequence of the UV radiation but is also due to the CO release. After irradiation of compound **5** and **10** the cytotoxicity slightly increased to 9 % and 7 %, respectively. These results reveal the potential of these compounds to act as photoCORMs. However, further investigations are necessary to clarify the mechanism. As expected, the effect on cell death was similar for compound **5** and **10** exhibiting different functionalities. This confirms that the amide‐functionalised moiety (alkyne or BODIPY) has no influence on the cytotoxicity and thus marks the crucial importance of the Ru^II^ dicarbonyl centre for the observed CO release in complex biological conditions.

## Conclusions

The application of prodrugs that generate toxic species (e.g., CO, NO, ^1^O_2_) upon exposure to electromagnetic radiation provides an excellent strategy to treat cancer diseases in a controlled way. Dicarbonyl ruthenium(II) complexes based on bipyridine ligands are suitable storage molecules for carbon monoxide to release the gas in a safe and controllable way. Two amide‐functionalised (4‐position) ruthenium(II) dicarbonyl bipyridyl complexes bearing an alkyne group **5** or a fluorescence tag **10**, consisting of a BODIPY (boron‐dipyrromethene) dye, were prepared for comparative studies on CO release, biological activity and cellular uptake. Concerning the latter, BODIPY‐based dyes feature a series of sophisticated properties for in vitro applications, inter alia, to investigate and to influence the uptake of labelled compounds to specific cell organelles.

The elucidation of the mechanism of CO release from photoCORMs is fundamental to extract necessary information about structural aspects influencing photoreactivity or ‐toxicity. The exposure to UV light induced a quick monodecarbonylation for both complexes, which was confirmed by ^13^C NMR and IR spectroscopy. A stepwise mechanism with two individual rate constants was determined by MCR‐ALS. The first rate constants were high and due to experimental setup they were considered lower limits only. In comparison, the second rate constants were determined at least one order of magnitude smaller.

Cell proliferation studies of both complexes were carried out using human embryonal kidney HEK293 and cancerous A431 cell lines. It was shown that reduction of HEK293 cell viability after exposure to UV light is associated with the bipyridyl dicarbonyl ruthenium complex as compound **5** and **10** exhibited similar results. In contrast, the cancerous cell line showed no significant reduction of viable cells. This result differs from the data described for photoCORMs based on Mn^I^ and Re^I^. Several factors must be taken into account to interpret the activity of different compounds in different cell lines. Cell lines are very complex and do not necessarily behave in the same way. So, the non‐cancerous HEK293 cell line is very sensitive to environmental conditions, whereas the cancerous cell line A431 is very robust and better able to adapt to changes.

Laser scanning confocal microscopy studies revealed that the fluorescent complex **10** was quickly taken up by A431 and HEK293 cells with an unspecific accumulation in mitochondria and the endoplasmic reticulum. Flow cytometry analysis indicates that after treatment with the Ru^II^ dicarbonyl complexes **5** and **10** after 10 min of UV irradiation, the cancerous A431 cells are already damaged after 4 h. The proportion of dead and apoptotic cells is comparable for both complexes, suggesting that the functionalisation of the bipyridine scaffold is of little if any importance. Further investigations are necessary to determine the mechanism of cell damage of the investigated photoCORMs. Future work in this direction will focus on the investigation of further cell lines and in particular on identification of cell organelles responsible for apoptosis.

## Experimental Section

### Materials and Methods

All syntheses were carried out using standard laboratory glassware. Prior to any light‐sensitive reactions, an adequate protection against light was ensured by wrapping the glassware in aluminium foil. All chemicals and solvents were purchased from the following suppliers and used without prior purification: ABCR, Across, Alfa Aesar, Applichem, Sigma‐Aldrich, TCI Europe and Thermo Fisher. Reaction progress was monitored by TLC using Merck silica gel 60 F_254_ plates and Macherey‐Nagel Polygram® SIL G/ UV254 plates with fluorescent indicator. Purification was performed by column chromatography (silica gel, particle size 0.035–0.07 mm, Acros Organics) or automated flash chromatography using a Biotage Isolera Four™ with a built‐in UV/Vis detector. IR spectra were recorded using a Fisher Scientific Nicolet iS5 FTIR spectrometer featuring a built‐in ATR module. The measurement was carried out in a range from 4000 to 400 cm^−1^ with a resolution of 0.482 cm^−1^. The background measurement was carried out on an empty ATR crystal. NMR spectra were recorded on two Agilent Technologies devices −400/54 Premium Shielded (400 MHz for ^1^H NMR, 101 MHz for ^13^C NMR, 128 MHz for ^11^B NMR, 376 MHz for ^19^F NMR) and 600/54 Premium Compact (600 MHz for ^1^H NMR, 151 MHz for ^13^C NMR, 192 MHz for ^11^B NMR, 564 MHz for ^19^F NMR). The samples were dissolved in a deuterated solvent purchased from Deutero. All measurement were carried out at 25 °C and the recorded NMR spectra were calibrated to the residual solvent signal^30^ (^1^H and ^13^C) or internal standards (^19^F: CFCl_3_, ^11^B: BF_3_.OEt_2_ in CDCl_3_). The chemical shift *δ* in parts per million (ppm) relative to tetramethylsilane, the signal multiplicity, the coupling constant *J* in Hertz, the number of cores and the assignment are given. The following abbreviations were used to describe the signal multiplicity: br=broad, d=doublet, dd=double doublet, m=multiplet, q=quartet, s=singlet, t=triplet, td=triplet of doublets. Mass spectra with electrospray ionisation (ESI) mode were carried out on a QuadroLC from Micromass. UV/Vis spectra were recorded using SPECORD 50 (Analytik Jena). The samples were transferred into Hellma quartz cuvettes of 1 cm optical path length and 3 mL volume. The measurements were carried out in a spectral range from 270 to 700 nm, with a spectral resolution of 1 nm. To determine the extinction coefficients, a 12.5 mm stock solution of the respective compound in DMSO was first prepared. For measurements in pure DMSO, 24 μL of the stock solution was mixed with 2976 μL DMSO. The resulting 100 μm solution was further diluted with pure DMSO as required. For measurements in 0.8 % (*v*/*v*) DMSO in water, 24 μL of the 12.5 mm stock solution were dissolved in 2976 μL water. The resulting 100 μm solution was diluted with a 0.8 % (v/v) DMSO/water mixture as required. Fluorescence spectra were recorded using LS 55 from PerkinElmer. The samples were transferred into Hellma quartz cuvettes of 1 cm optical path length and 3 mL volume. The measurements were carried out in a spectral range from 500 to 700 nm, with an excitation wavelength of 509 nm and spectral resolution of 0.5 nm. Sample preparation was carried out in analogy to UV/Vis spectroscopy.

Photolysis experiments were conducted using a temperature‐controlled CCP‐ICH2 photoreactor from Luzchem fitted with 16 deuterium lamps with emission wavelengths centred at 350 nm (full width at half maximum, FWHM±25 nm; *E*_v_≈6 mW cm^−2^). A quartz fluorescence cuvette from Hellma (*V=*3 mL; *d*=1 cm) was used as reaction vessel. The extinction coefficients with 3157 and 11,100 m ^−1^⋅cm^−1^ at 350 nm are well‐suited to induce an efficient photo‐decarbonylation reaction.

**Ferrioxalate actinometry**: Ferrioxalate actinometry was used to determine how much light is absorbed through a photoreaction.^31^ Experiments were performed as described in the literature.10a A photon flow (*I*_abs_) of about 3.8×10^−8^ Einstein s^−1^ was determined. The reaction rate of a photoreaction is best described by Equation (1). Conditional rate constants can be derived were (pseudo)first order kinetic is observed.18a, 18b

(1) $$-\frac{dc}{dt}=\Phi I_{abs}=kc$$

where *c* represents concentration, *t* time, *I*_abs_ the absorbed light intensity (photon flux), and *Φ* the quantum yield.

**CO/CO_2_ quantification**: 10 mL of 0.314 mm solution of complex **5** in DMSO/water (3:1, v/v) was added to a 60 mL reaction vessel together with 200 μL of pure CH_4_ as internal reference. The solution was irradiated using a LED lamp (390 nm, photon flux of 0.35±0.02 μE s^−1^) for 20 h under N_2_ atmosphere. Gas chromatograms were recorded using a Varian CP‐3800 gas chromatograph with helium as the carrier gas and a 3 m×2 mm carboxen‐1000, 60/80 column. The gas flow was set to 20 mL min^−1^. The oven was operated isothermally at 100 °C. The 100 μL gas samples of the headspace were injected using a Hamilton (1825 RN) gastight microliter syringe. The gases were detected using a thermal conductivity detector (Varian) operated at 150 °C. Calibrations were performed by the injection of known quantities of pure gases (CO or CO_2_) diluted in a reaction vessel. 200 μL of pure CH_4_ was used as internal reference to calculate the dilution factor.

### Cell experiments

Human A431 (skin epidermoid carcinoma) and HEK293 (human embryonic kidney) cell lines were purchased from CLS (Cell Lines Service GmbH, Eppelheim, Germany) and DSMZ Leibniz Institute (DSMZ—German Collection of Microorganisms and Cell Cultures). The cells were cultivated in Dulbecco modified Eagle medium (DMEM), with or without the phenol red indicator and supplemented with heat inactivated 10 % fetal bovine serum (FBS), penicillin and streptomycin. All ingredients of the cell medium were purchased from BIOCHROM or Thermo Fisher Scientific. Cells were cultivated at 37 °C in 5 % CO_2_.

The MTS assay cell experiments were performed in Cellstar transparent flat bottom 48‐well plates. The cells were seeded with a density of 10 000 cells per well. Each well contained 200 μL of media. Cells were incubated for 24 h at 37 °C in a humidified atmosphere enriched with 5 % CO_2_.

The photoCORMs **5** and **10** were added after 24 h. A freshly prepared 12.5 mm stock solution of **5** and **10** in DMSO was diluted with cell medium depending on the final concentration. The final concentrations of **5** and **10** were adjusted to 25/50/60/75/100 μm and 3.12/6.25/12.5/25/50 μm by adding 2.4 μL DMSO solution of **5** and **10** and 97.6 μL cell medium. The final concentration of DMSO in each well was then 0.8 % (v/v). Two negative controls were used; either DMSO‐containing cell medium (0.8 % (v/v)) or just cell medium with final volumes of 300 μL per well. The outer wells were filled with phosphate buffer (PBS) to prevent the samples from evaporating.

24 h after the treatment of the cells with the respective complex, the cells were irradiated at 350 nm for 10 min using the Luzchem photoreactor CCP‐ICH2. The cell medium was changed immediately after the irradiation—the old medium was removed and replaced by a fresh, DMSO‐ and photoCORM‐free one. The well plates were then stored in the incubator. Cells viability was measured using an MTS assay 24 or 48 h after the irradiation.

**Cytotoxicity assay**: Cell viability was determined using the commercially available Promega CellTiter 96® aqueous one solution cell proliferation assay MTS kit. 24 and 48 h after the irradiation, 50 and 60 μL of the MTS reagent were added to each well and left in the incubator for further 1–2 h. The formed formazan was detected at 492 nm using the TECAN microplate reader Sunrise™.

**Intracellular uptake and colocalisation studies**: Cellular uptake and organelle colocalisation of compound **10** was evaluated by laser scanning confocal microscopy (LSCM). Compound **10** (10 or 50 μm) was incubated for 0.5 h and 4 h at 37°C in 5 % CO_2_ atmosphere. Compound **10** was detected using the green colour channel (*λ*_ex_=485 nm, filter: 490–590 nm). Cells were visualised using an Olympus FV10‐ASV confocal laser scanning microscope (Olympus Czech group Ltd., Prague, Czech Republic) equipped with a 60x oil objective. Image analysis and level of the colocalization were determined using ImageJ software. Pearson correlation coefficients (PCC) were calculated using the same software.

**Mitochondria, endoplasmic reticulum and colocalization**: The cells were seeded with a density of 3×10^5^ cells per well on 4 chamber glass bottom dishes (35 mm *x* 20 mm bottom well, 0.13–0.16 mm, Bio‐Port Europe s.r.o., Prague, Czech Republic). To the respective cells were added 10 μm or 50 μm (in 0.1 % or 0.5 % DMSO) of compound **10** and incubated for 0.5 h or 4 h at 37 °C. Then, the cells were washed with Dulbecco's modified PBS (DPBS) and labelled with a mitochondria selective probe, MitoTracker® (molecular probes, Thermo Fisher Scientific, Czech Republic) using 500 nm and for the endoplasmic reticulum ER‐Tracker™ Blue‐White DPX dyes for live‐cell imaging (Molecular Probes, Thermo Fisher Scientific, Czech Republic) in a final concentration of 1 μm. The cell nuclei were labelled using Hoechst 33342 dye and cell membrane using Cell Mask™ Deep Red marker (Thermo Fischer Scientific, Czech Republic). The cells were labelled for 15 min at 37 °C in 5 % CO_2_, then the cells were washed twice with DPBS and visualized.

**Flow cytometry**: For flow cytometry the cells were seeded in 1 mL of media into 24‐well plates (TPP, Czech Republic) at density 1×10^5^ cells per well. The cells were incubated with the compounds for 0.5 h and then irradiated for 10 min with UV light. The final concentration of compound **5** and **10** were 10 μm in 0.1 % DMSO. Medium was replaced by fresh one after the irradiation and the cells were incubated another 4 h before analysis. Then, the cells were detached from the cultivation plate by using 0.05 % trypsin (Thermo Fisher Scientific, Czech Republic) and washed with PBS, and resuspended in 200 μL 1x binding buffer (Bb) (10x 0.1 m Hepes/NaOH, 1.4 m NaCl, 25 mm CaCl_2_ pH 7.4) and incubated for 10 min at room temperature with Annexin V‐APC (ebioscience, Europe) diluted in Bb (5 μL of Annexin V‐APC in 195 μL Bb). Subsequently, the cells were washed and resuspended in Bb with lethal marker 7‐aminoactinomycin (7AAD) (1 μg mL^−1^) (Invitrogen, USA) and incubated for 15 min at room temperature. Then, the fluorescent signals were evaluated using BD FACSverse™ flow cytometer (BD Bioscience, San Jose, USA) and the obtained data were analysed using flowJo software (Ashland, Oregon‐based FlowJo LLC, USA) as medians of fluorescence intensity. The experiments were prepared in four replicates. The statistical analysis of data were performed using Graph Pad Prism software 5.1 (Dunnett post test as part of one‐way ANOVA analysis).

**Time dependent cellular uptake**: The cells were seeded with a density of 3×10^5^ cells per well on 4‐chamber glass bottom dishes (35 mm×20 mm bottom well, 0.13–0.16 mm, Bio‐Port Europe s.r.o., Prague, Czech Republic). Compound **10** was added at final concentration of 10 μm containing 0.1 % DMSO and the uptake was visualised by LSCM after 4, 8, 12, 16, 20 and 24 min.

### Synthesis

**4′‐Methyl‐*N*‐(propargyl)‐(2,2′‐bipyridine)‐4‐carboxamide (4)**: 60 mg (0.28 mmol; 1 equiv.) of 4′‐methyl‐(2,2′‐bipyridine)‐4‐carboxylic acid, 64 mg (0.34 mmol; 1.2 equiv.) of EDC⋅HCl, 45 mg (0.34 mmol; 1.2 equiv.) of HOBt and 48 μL (0.28 mmol, 1 equiv.) of DIPEA were dissolved in 5 mL of anhydrous DMF and stirred under argon for 10 min. 18 μL (0.28 mmol, 1 equiv.) of propargylamine and 48 μL (0.28 mmol, 1 equiv.) of DIPEA in 5 mL of anhydrous DMF were then added and the reaction mixture was stirred at room temperature overnight. After removal of the solvent, the crude product was purified by means of column chromatography (silica, methanol/chloroform 1:5 (*v*/*v*)) and obtained **4** as a colourless solid. Yield: 43 mg (61 %). ^1^H NMR (400 MHz, CD_3_CN): *δ*=8.76 (dd, ^3^ *J*=5.0 Hz, ^4^ *J*=0.9 Hz, 1 H), 8.71 (s, 1 H), 8.54 (d, ^3^ *J=*4.8 Hz, 1 H), 8.27 (s, 1 H), 7.74–7.61 (m, 2 H), 7.25 (d, ^3^ *J*=4.9 Hz, 1 H), 4.16 (dd, ^3^ *J*=5.7 Hz, ^4^ *J*=2.5 Hz, 2 H), 2.50 (t, ^4^ *J*=2.5 Hz, 1 H), 2.44 ppm (s, 3 H); ^13^C NMR (101 MHz, CD_3_CN): *δ*=166.2 (C=O), 158.0, 156.0, 151.0 (C_ar_), 150.2 (C_ar_), 149.6, 143.3, 126.2 (C_ar_), 122.5 (C_ar_), 122.1 (C_ar_), 119.1 (C_ar_), 81.0, 72.0, 29.7, 21.3 ppm. MS (ESI) *m*/*z* (%): 252 (100) [*M*+H]^+^ (calcd for C_15_H_14_N_3_O^+^: 252.29)

***trans*** **‐Cl,** ***cis*** **‐CO[Ru(4)Cl_2_(CO)_2_] (5)**: Under exclusion of light and under argon, 31 mg of (0.12 mmol, 1 equiv.) **4** and 28 mg of [RuCl_2_(CO)_2_]_*n*_ were suspended in 6 mL of degassed methanol and stirred and reflux for 30 min. After cooling, the mixture was concentrated under reduced pressure to approximately 2 mL and placed in the refrigerator overnight. The formed precipitate was filtered and washed with 8 mL cold methanol. The precipitate was collected and dried in vacuo to obtain a yellow solid. Yield: 35 mg (59 %). ^1^H NMR (400 MHz, CD_3_CN): *δ*=9.27 (d, ^3^ *J*=5.8 Hz, 1 H), 9.00 (d, ^3^ *J*=5.7 Hz, 1 H), 8.72 (d, ^4^ *J*=1.7 Hz, 1 H), 8.45 (s, 1 H), 7.98 (dd, ^3^ *J*=5.7 Hz, ^4^ *J*=1.8 Hz, 1 H), 7.77 (s, 1 H), 7.60 (d, ^3^ *J*=5.1 Hz, 1 H), 4.22 (dd, ^3^ *J*=5.5 Hz, ^4^ *J*=2.5 Hz, 2 H), 2.61 (s, 3 H), 2.56 ppm (t, ^4^ *J*=2.6 Hz, 1 H); ^1^H NMR (400 MHz, [D_6_]DMSO): *δ*=9.59 (t, ^3^ *J*=5.6 Hz, 1 H), 9.37 (d, ^3^ *J*=5.7 Hz, 1 H), 9.08 (d, ^3^ *J*=5.7 Hz, 1 H), 9.01 (s, 1 H), 8.74 (s, 1 H), 8.11 (d, ^3^ *J*=5.7 Hz, 1 H), 7.71 (d, ^3^ *J*=5.7 Hz, 1 H), 4.19 (d, ^3^ *J*=3.1 Hz, 2 H), 3.27 (s, 1 H), 2.62 ppm (s, 3 H); ^13^C NMR (101 MHz, [D_6_]DMSO): *δ*=196.3 (C≡O), 196.1 (C≡O), 162.9 (C=O), 155.3 (C_ar_), 153.9 (C_ar_), 153.4 (C_ar_), 152.8 (C_ar_), 152.5 (C_ar_), 144.4 (ar), 128.9 (C_ar_), 125.3 (C_ar_), 125.3 (C_ar_), 121.5 (C_ar_), 80.3, 73.9, 29.0, 20.9 ppm; IR (ATR): $\tilde{\nu }$ =3292 (m, alkyne), 2073 (vs., C≡O), 2016 (vs., C≡O), 1672 cm^−1^ (s, C=O); UV/Vis (0.8 % (v/v) DMSO/H_2_O): *λ*_max_ (*ϵ*)=313 (12,852), 324 nm (13,502 L mol^−1^⋅cm^−1^). Elemental analysis calcd (%) for C_17_H_13_Cl_2_N_3_O_3_Ru: C 42.60, H 2.73, N 8.77, found: C 42.11, H 3.01, N 8.88

**4,4‐Difluoro‐1,3,5,7‐tetramethyl‐8‐(4‐(azidomethyl)phenyl)‐4‐bora‐3a,4a‐diaza‐*s*‐indacene (7)**: 439 mg (1.12 mmol; 1 equiv.) of 6, 83 mg (1.28 mmol; 1.14 equiv.) of NaN_3_ and 15 mg (0.1 mmol; 0.1 equiv.) of KI were dissolved in 7 mL of DMF. The solution was stirred at 45 °C overnight. After cooling down, 100 mL of water was added to the reaction mixture and extracted with CHCl_3_ (3×40 mL). The organic layer was dried over Na_2_SO_4_, the solution was evaporated in vacuo and purified by column chromatography (silica, petrolether/EtOAc 10:1 (*v*/*v*), *R*_f_=0.39 (CHCl_3_)) to afford **7** as a red solid. Yield: 498 mg (quant.). ^1^H NMR (400 MHz, CDCl_3_): *δ*=7.45 (d, ^3^ *J*=8.4 Hz, 2 H), 7.32 (d, ^3^ *J*=8.4 Hz, 2 H), 5.98 (s, 2 H), 4.44 (d, ^3^ *J*=5.9 Hz, 2 H), 2.56 (s, 6 H), 1.37 ppm (s, 6 H); ^13^C NMR (101 MHz, CDCl_3_): *δ*=155.7, 141.0, 136.0, 135.1, 131.1, 129.0, 128.6, 121.3, 121.3, 54.3, 14.6, 14.4 ppm; ^11^B NMR (128 MHz, CDCl_3_): *δ*=0.8 ppm (t, ^1^ *J*=33.1 Hz); ^19^F NMR (376 MHz, CDCl_3_): *δ*=−146.4 ppm (q, ^1^ *J*=32.5 Hz). MS (ESI) *m*/*z* (%): 380 (100) [*M*+H]^+^ (calcd for C_20_H_21_BF_2_N_5_^+^: 380.22); UV/Vis (CH_3_CN): *λ*_max_ (*ϵ*)=490 nm (15900 L mol^−1^⋅cm^−1^); fluorescence (CH_3_CN): *λ*_ex_=490 nm; *λ*_em_=509 nm.

**4’‐Methyl‐2,2’‐bipyridine‐4‐carboxylic acid‐*N*‐4‐(4,4‐difluoro‐1,3,5,7‐tetramethyl‐4‐bora‐3a,4a‐diaza‐*s*‐indacene‐8‐yl)benzylamide (9)**: Under argon, 100 mg (0.47 mmol; 1 equiv.) of 4′‐methyl‐2,2′‐bipyridine‐4‐carboxylic acid (**1 b**), 192.6 mg (0.93 mmol; 2 equiv.) of DCC and 69 mg (0.51 mmol; 1.1 equiv.) of HOBt were dissolved in 5 mL of DMF and stirred overnight at room temperature. In a different flask and under argon, 177 mg (0.47 mmol; 1 equiv.) of compound **7** and 134.7 mg (0.51 mmol; 1.1 equiv.) of triphenylphosphine were dissolved in 5 mL of DMF and stirred at room temperature overnight. Afterwards, the contents of both flasks were combined and the resulting solution was stirred at room temperature for another 24 h. Upon removing the solvent, the crude product was purified by flash chromatography (silica, ethyl acetate→ethyl acetate/chloroform 1:1 (v/v)) and obtained as a bright‐red solid. Yield: 120 mg (47 %). ^1^H NMR (400 MHz, CDCl_3_): *δ*=8.73 (d, ^3^ *J*=5.0 Hz, 1 H), 8.66 (s, 1 H), 8.44 (d, ^3^ *J*=5.0 Hz, 1 H), 8.22 (s, 1 H), 7.75 (dd, ^3^ *J*=5.0 Hz, ^4^ *J*=1.7 Hz, 1 H), 7.46–7.33 (m, 3 H), 7.20 (d, ^3^ *J*=8.1 Hz, 2 H), 7.11 (d, ^3^ *J*=4.9 Hz, 1 H), 5.91 (s, 2 H), 4.71 (d, ^3^ *J*=5.9 Hz, 2 H), 2.49 (s, 6 H), 2.40 (s, 3 H), 1.32 ppm (s, 6 H); ^13^C NMR (101 MHz, CDCl_3_): *δ*=165.8 (C=O), 157.0, 155.5, 155.0, 150.1, 148.9, 148.6, 143.1, 142.4, 141.3, 139.1, 134.2, 131.4, 128.4, 128.3, 125.3, 122.3, 121.7, 121.3, 117.6, 43.6, 21.2, 14.6 ppm; ^11^B NMR (192 MHz, CDCl_3_): *δ*=−1.9 ppm (t, ^1^ *J*=33.1 Hz); ^19^F NMR (564 MHz, CDCl_3_): *δ*=−148.8 ppm (q, ^1^ *J*=32.5 Hz). MS (ESI^+^) *m*/*z* (%): 550 (100) [*M*+H]^+^ (calcd for C_32_H_31_BF_2_N_5_O^+^: 550.43).

***trans*** **‐Cl,*cis*‐CO[Ru(9)Cl_2_(CO)_2_] (10)**: Under exclusion of light and under argon, 57 mg (0.1 mmol, 1 equiv.) of compound **9** and 47 mg of [RuCl_2_(CO)_2_]_*n*_ were suspended in 8 mL of degassed methanol and stirred and reflux for 2 h. After cooling down, the reaction flask was deposited in the refrigerator for 1 h. The precipitate was filtered and washed with 8 mL of cold methanol. The filtrate was discarded. The precipitate was collected and dried in vacuo to obtain a red solid. Yield: 55 mg (68 %). ^1^H NMR (400 MHz, CDCl_3_): *δ*=9.12 (d, ^3^ *J*=5.6 Hz, 1 H), 8.91 (d, ^3^ *J*=5.5 Hz, 1 H), 8.56 (s, 1 H), 8.09 (s, 1 H), 7.97 (d, ^3^ *J*=5.6 Hz, 1 H), 7.68 (t, ^3^ *J*=5.4 Hz, 1 H), 7.51 (d, ^3^ *J*=7.9 Hz, 2 H), 7.42 (d, ^3^ *J*=5.6 Hz, 1 H), 7.30 (d, ^3^ *J*=7.8 Hz, 2 H), 5.97 (s, 2 H), 4.73 (d, ^3^ *J*=5.8 Hz, 2 H), 2.55 (s, 6 H), 2.50 (s, 3 H), 1.38 ppm (s, 6 H); ^1^H NMR (400 MHz, [D_6_]DMSO): *δ*=9.76 (t, ^3^ *J*=6.1 Hz, 1 H), 9.40 (d, ^3^ *J*=5.7 Hz, 1 H), 9.13–9.04 (m, 2 H), 8.31 (s, 1 H), 8.19 (d, ^3^ *J*=5.7 Hz, 1 H), 7.72 (d, ^3^ *J=*5.6 Hz, 1 H), 7.57 (d, ^3^ *J*=7.9 Hz, 2 H), 7.36 (d, ^3^ *J*=7.8 Hz, 2 H), 6.17 (s, 2 H), 4.74 (d, ^3^ *J*=5.9 Hz, 2 H), 2.62 (s, 3 H), 2.44 (s, 6 H), 1.36 ppm (s, 6 H); ^13^C NMR (101 MHz, [D_6_]DMSO): *δ*=196.3 (C≡O), 196.1 (C≡O), 163.4 (C=O), 155.3, 154.9, 154.0, 153.4, 152.8, 152.6, 144.8, 142.7, 141.9, 139.7, 132.6, 130.8, 128.9, 127.9, 127.7, 125.4, 125.3, 121.7, 121.4, 42.6, 20.9, 14.2, 14.2 ppm; ^11^B NMR (128 MHz, [D_6_]DMSO): *δ*=0.6 ppm (t, ^1^ *J*=33.2 Hz); ^19^F NMR (376 MHz, [D_6_]DMSO): *δ*=−143.7 ppm (q, ^1^ *J*=31.6 Hz); MS (ESI^−^) *m*/*z* (%): 776 (100) [*M*−H]^−^ (calcd for C_34_H_29_BCl_2_F_2_N_5_O_3_Ru^−^: 776.41); IR (ATR): $\tilde{\nu }$ =3307 (m); 2079 (vs., C≡O); 2008 (vs., C≡O), 1667 cm^−1^ (s, C=O); UV/Vis (0.8 % (*v*/*v*) DMSO/H_2_O): *λ*_max_ (*ϵ*)=313 (19,267), 324 (19,300), 509 nm (36 800 L mol^−1^⋅cm^−1^); fluorescence (0.8 % (*v*/*v*) DMSO/H_2_O): *λ*_ex_=509 nm; *λ*_em_=536 nm.

## Conflict of interest

The authors declare no conflict of interest.

## Supporting information

As a service to our authors and readers, this journal provides supporting information supplied by the authors. Such materials are peer reviewed and may be re‐organized for online delivery, but are not copy‐edited or typeset. Technical support issues arising from supporting information (other than missing files) should be addressed to the authors.

Supplementary
