# Supplementary material for: Light‐Activated Carbon Monoxide Prodrugs Based on Bipyridyl Dicarbonyl Ruthenium(II) Complexes
Source: Chemistry. 2020 Aug 13;26(48):10992–1006. doi: 10.1002/chem.202002139 (PMC7496190; doi:10.1002/chem.202002139)
Supplement: Supplementary file 1 — Supplementary [file CHEM-26-10992-s001.pdf]

Chemistry–A European Journal

Supporting Information

Light-Activated Carbon Monoxide Prodrugs Based on Bipyridyl Dicarbonyl Ruthenium(II) Complexes

Stepan Geri,[a] Tereza Krunclova,[b] Olga Janouskova,[b] Jiri Panek,[c] Martin Hruby,[c] Daniel Hernández-Valdés,[d] Benjamin Probst,[d] Roger A. Alberto,[d] Constantin Mamat,[a] Manja Kubeil,*[a] and Holger Stephan*[a]

Table of Contents:

NMR spectra of ligands **4**, **7**, **9** and complexes **5** and **10**	S1, S2, S4, S5-S8, S10, S11
^{13}C NMR spectra of irradiated **5** and **10**	S14
IR spectra of complexes **5** and **10** and spectra of irradiated **5** and **10**	S3, S13, S15
ESI mass spectra of ligands **7**, **9** and complex **10**	S6, S9, S12
MCR-ALS spectra of **5** and **10**	S16, S17
GC-TCD mass spectra	S18
Cellular localisation studies of compound **10** in A431 and HEK293 cells	S19, S20
Flow cytometry analysis of compounds **5** and **10** in A431 cells before and after irradiation	S21

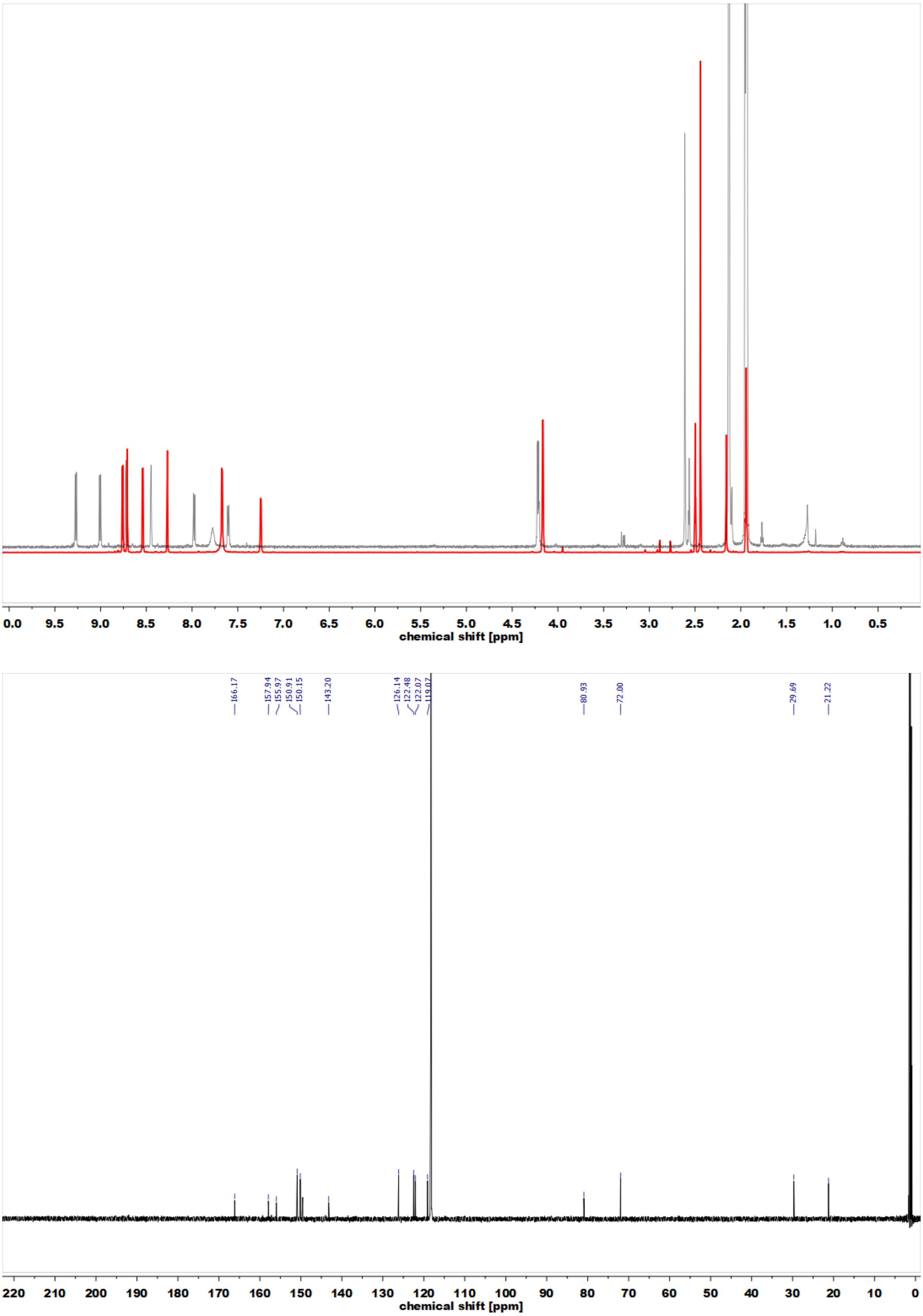

Figure S1. ^{1}H NMR (top) spectra of ligand **4** (red) and complex **5** (grey) and ^{13}C NMR (bottom) spectrum of ligand **4** measured in CD_3CN at 25 °C.

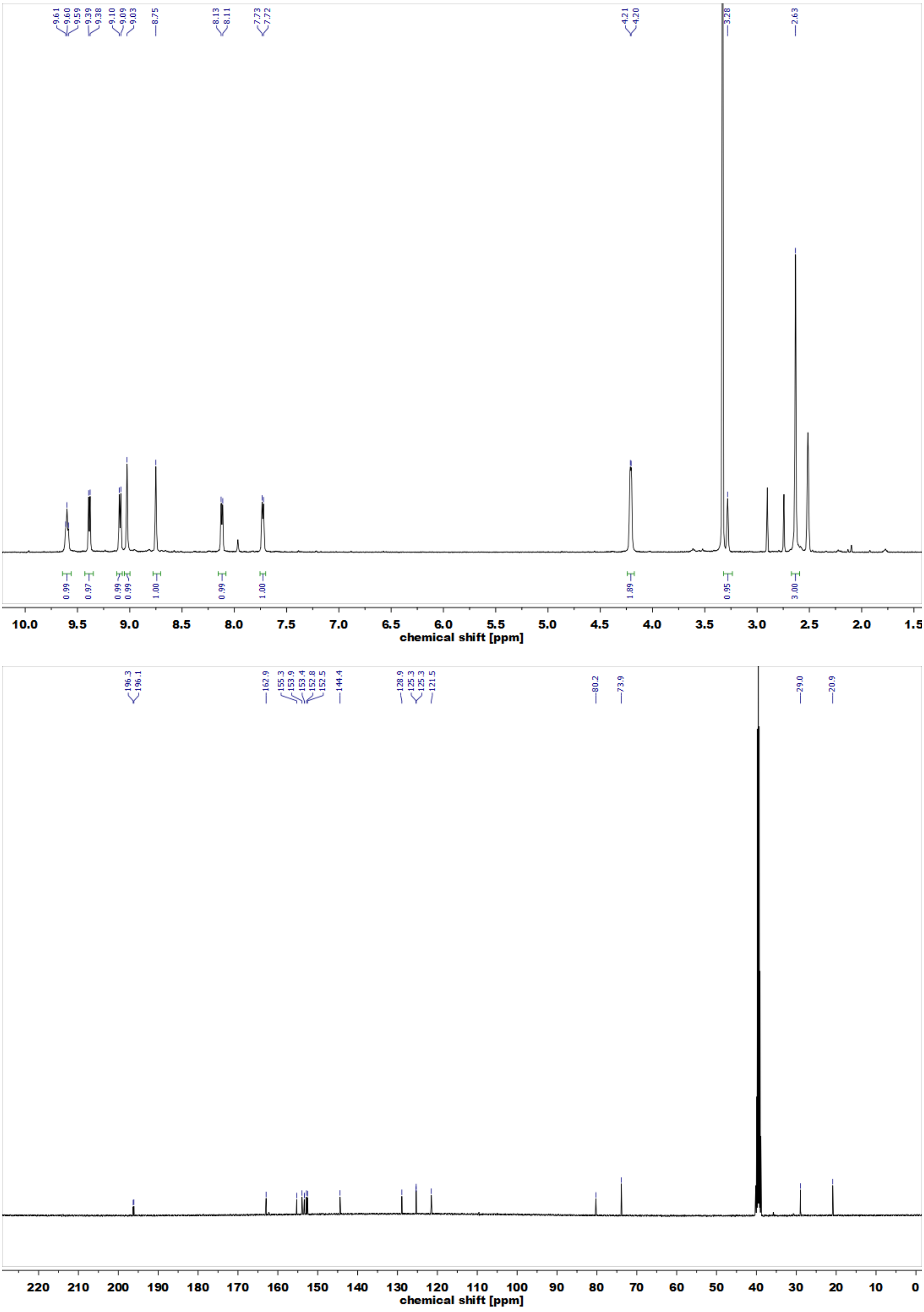

Figure S2. ^{1}H (top) and ^{13}C NMR (bottom) spectra of complex **5** measured in DMSO-d_6 at 25 °C.

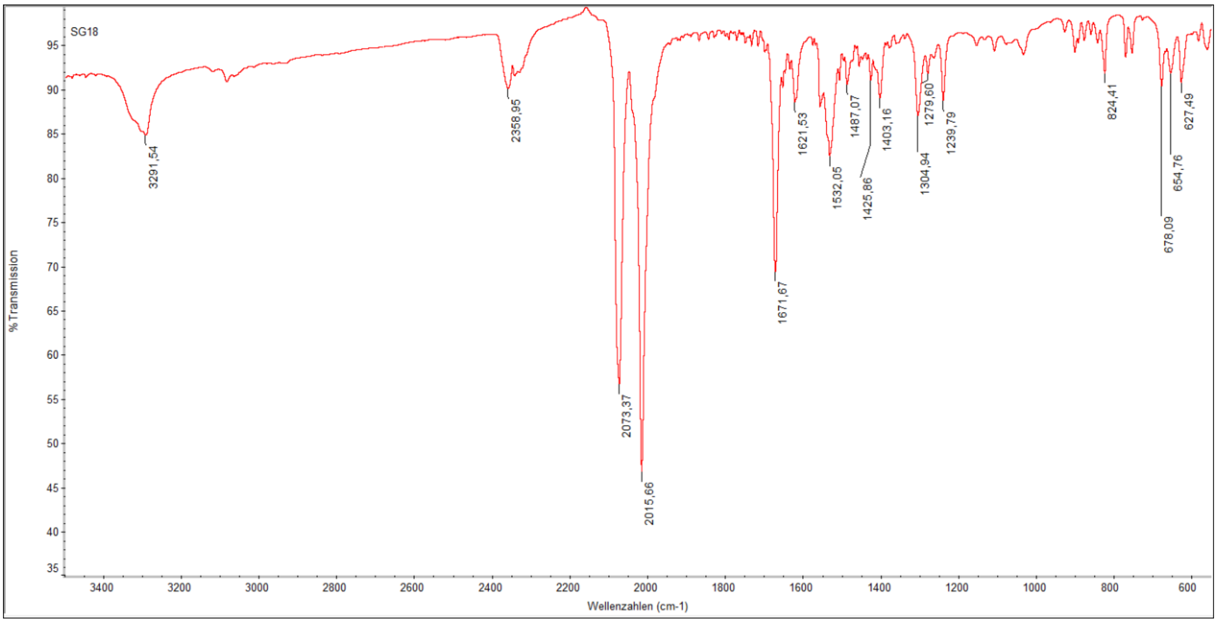

Figure S3. FT-IR spectrum of complex **5**.

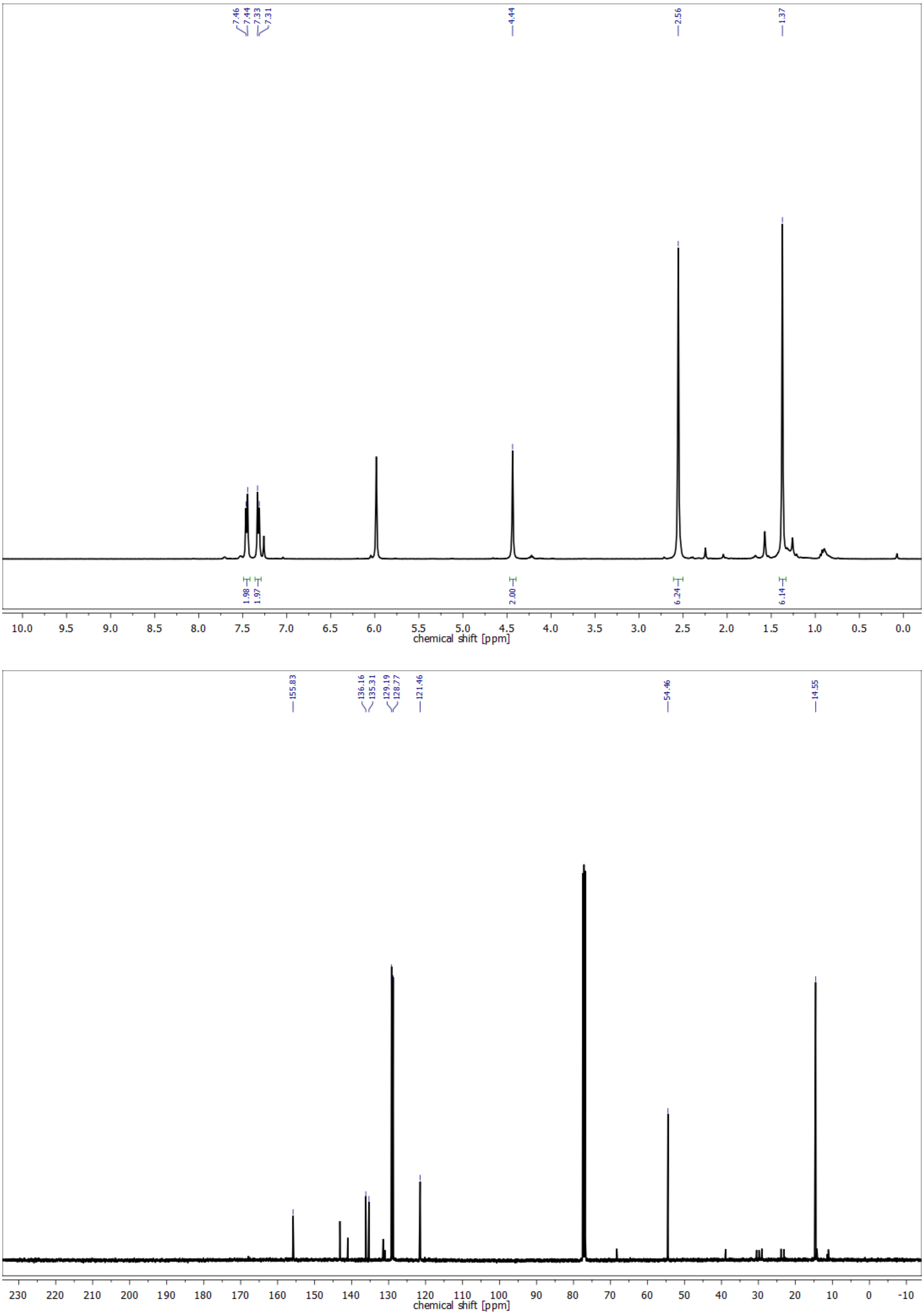

Figure S4. ^{1}H (top) and ^{13}C NMR (bottom) spectra of ligand **7** measured in $CDCl_3$ at 25 °C.

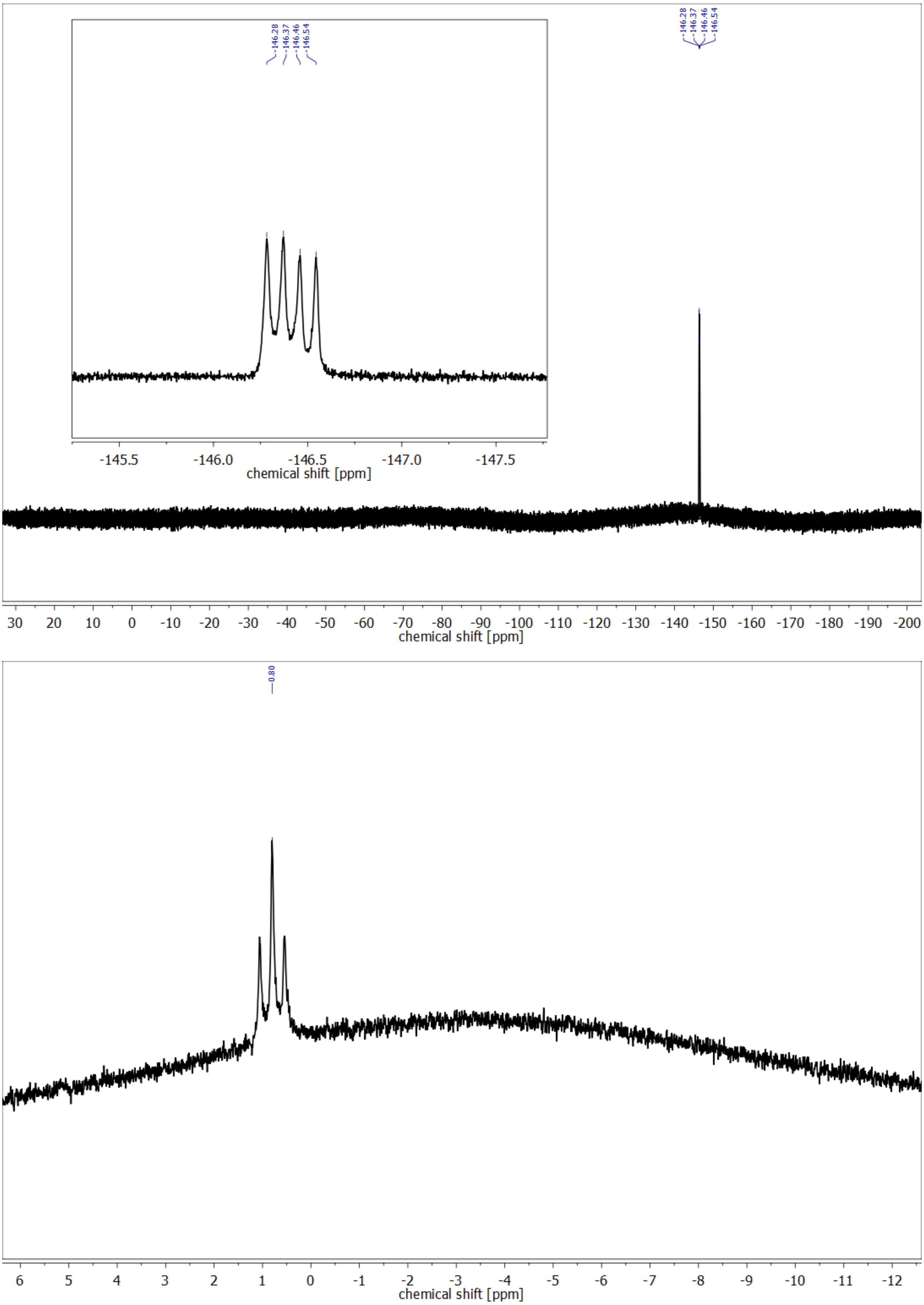

Figure S5. ^{19}F (top) and ^{11}B (bottom) NMR spectra of ligand **7** measured in $CDCl_3$ at 25 °C.

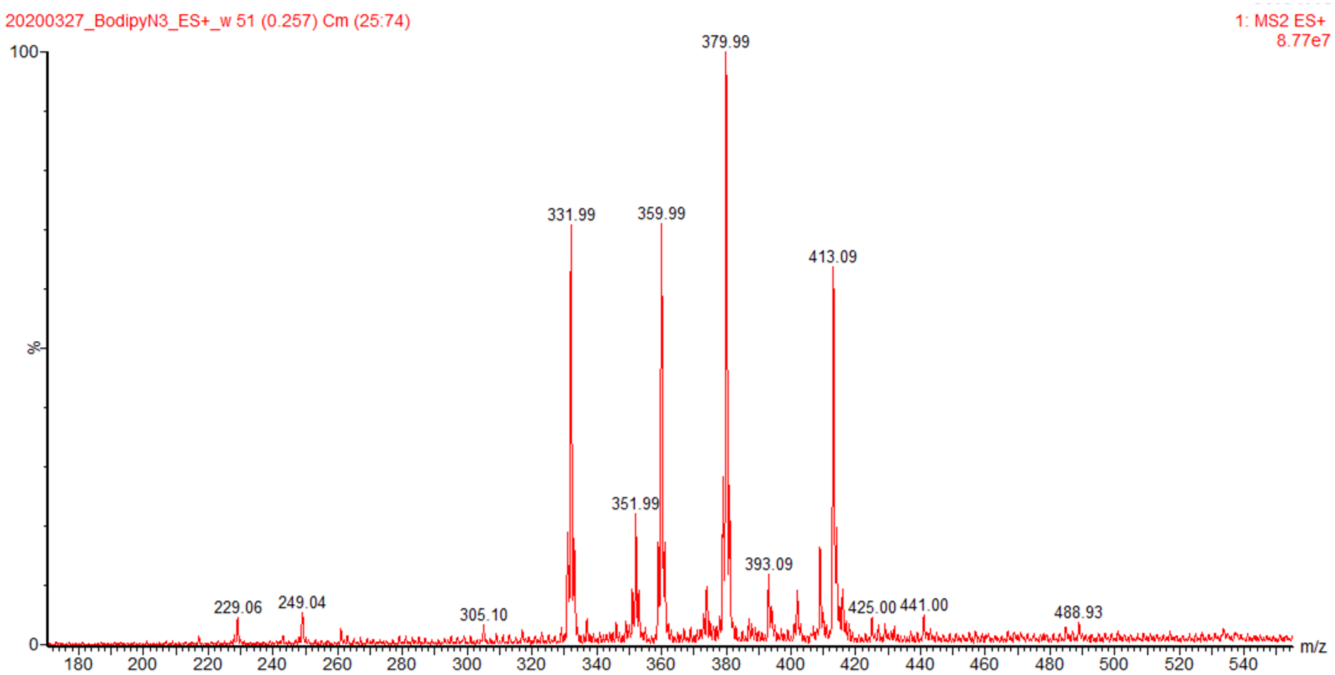

Figure S6. ESI (positive mode) mass spectrum of compound **7** in CH_3CN.

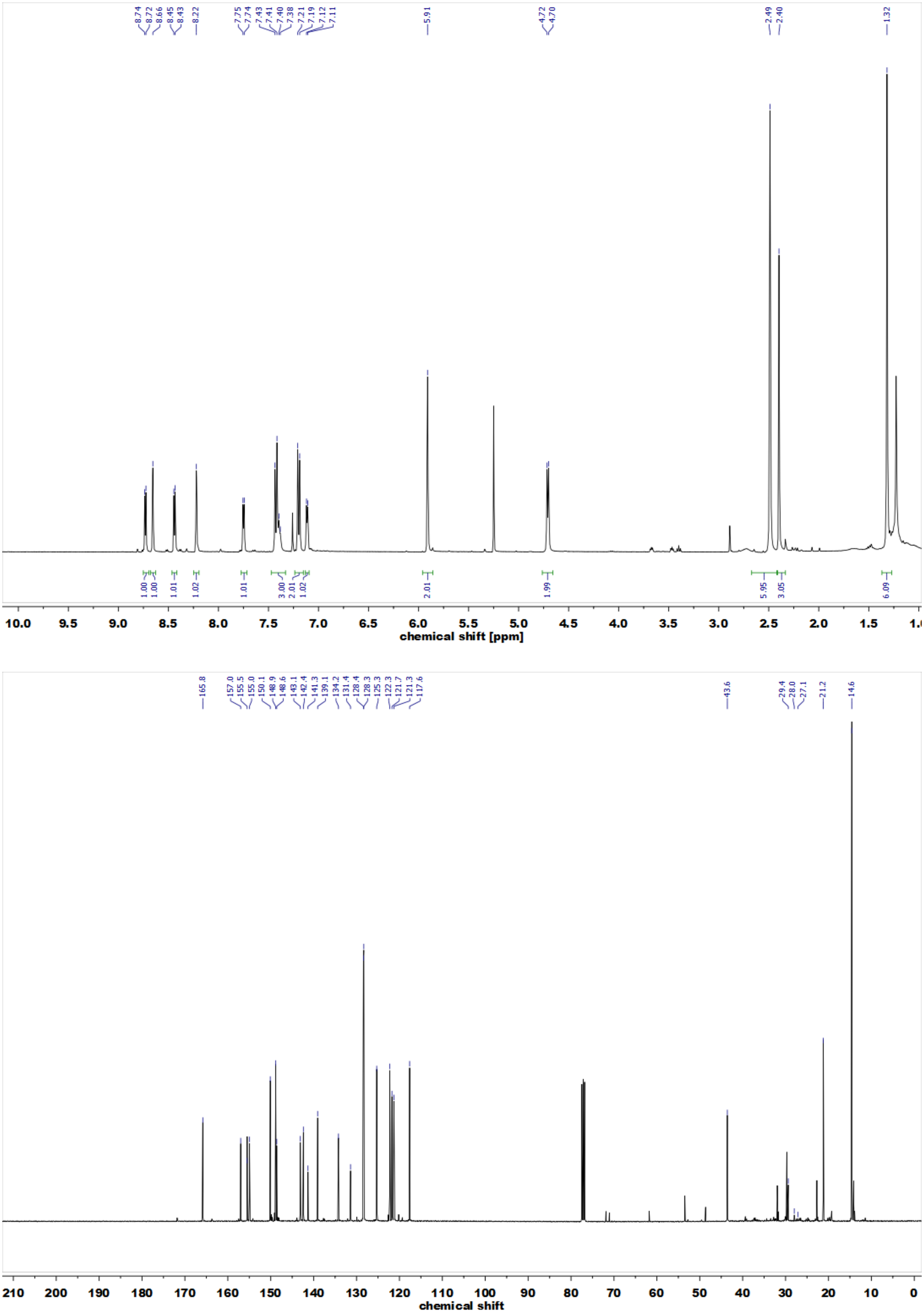

Figure S7. ^{1}H (top) and ^{13}C NMR (bottom) spectra of ligand **9** measured in $CDCl_3$ at 25 °C.

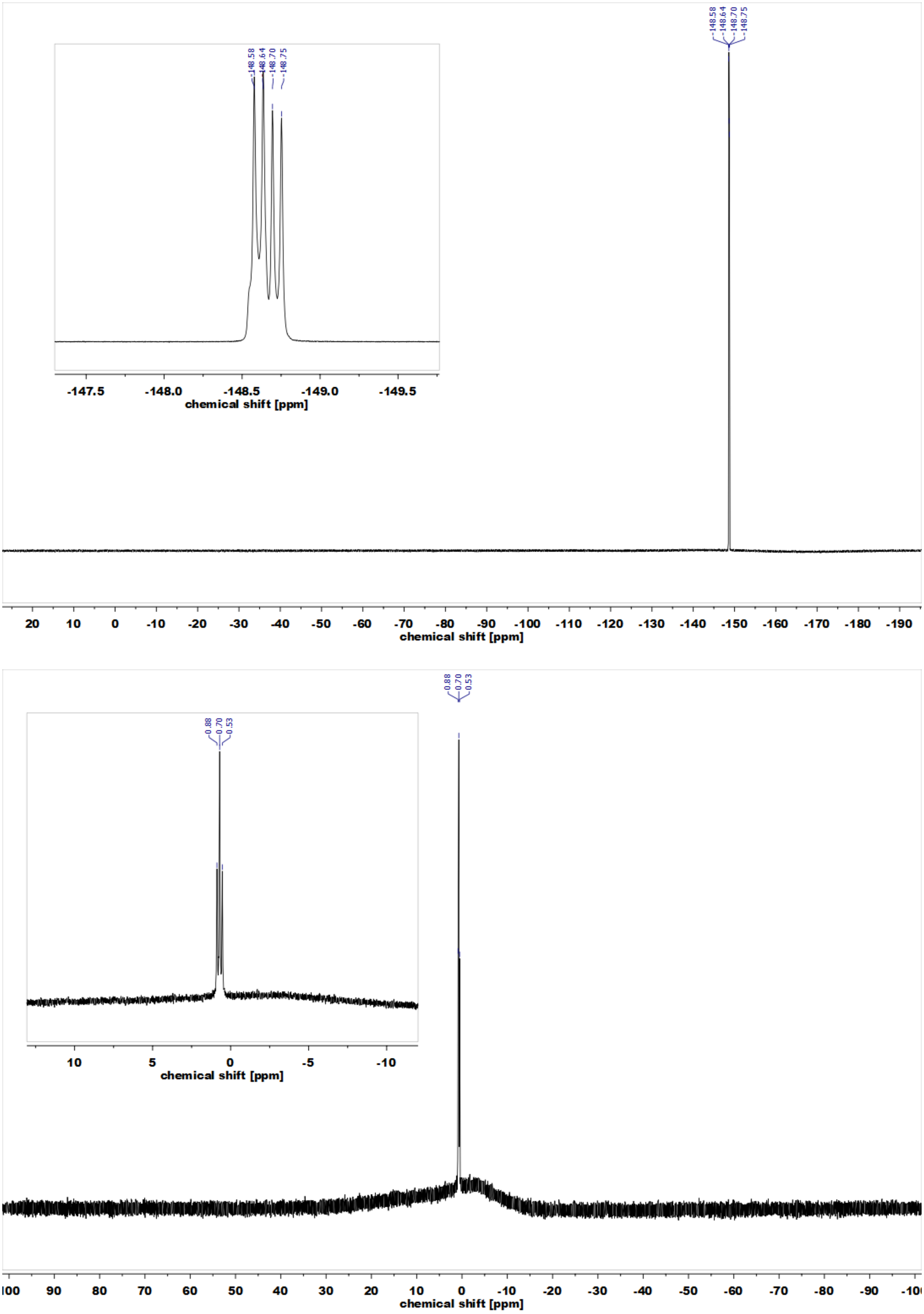

Figure S8. ^{19}F (top) and ^{11}B (bottom) spectra of ligand **9** measured in $CDCl_3$ at 25 °C.

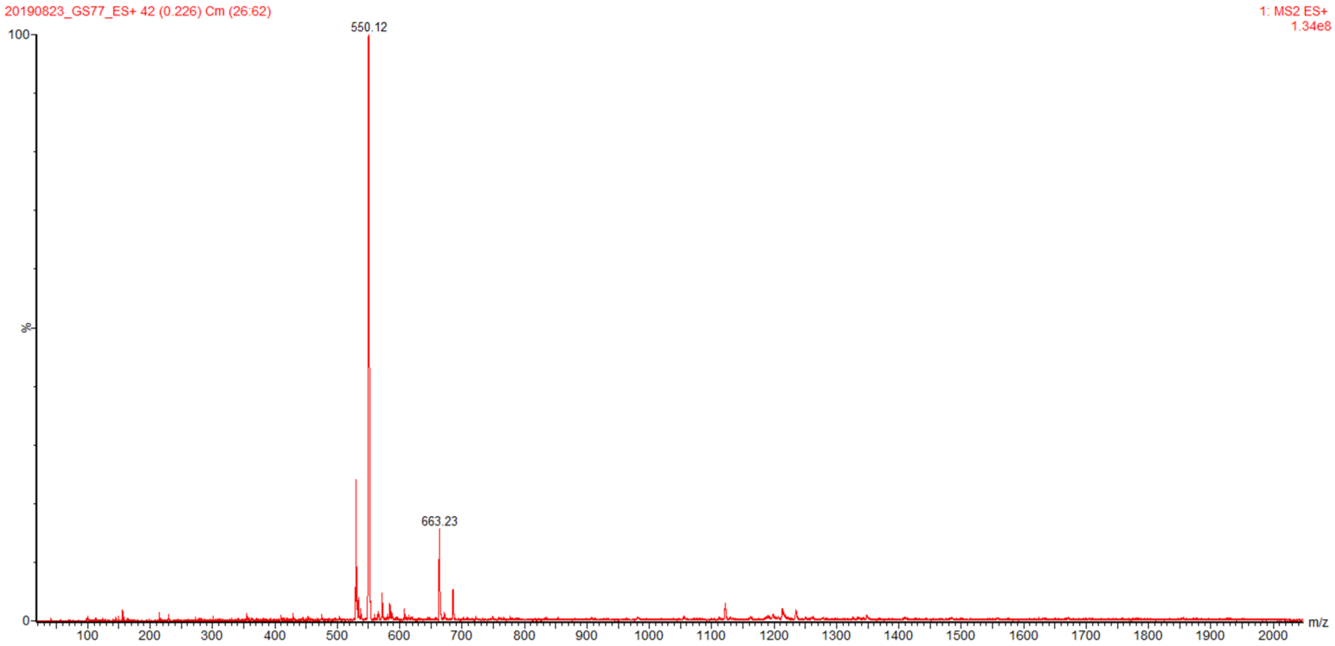

Figure S9. ESI (positive mode) mass spectrum of compound **9** in CH_3CN.

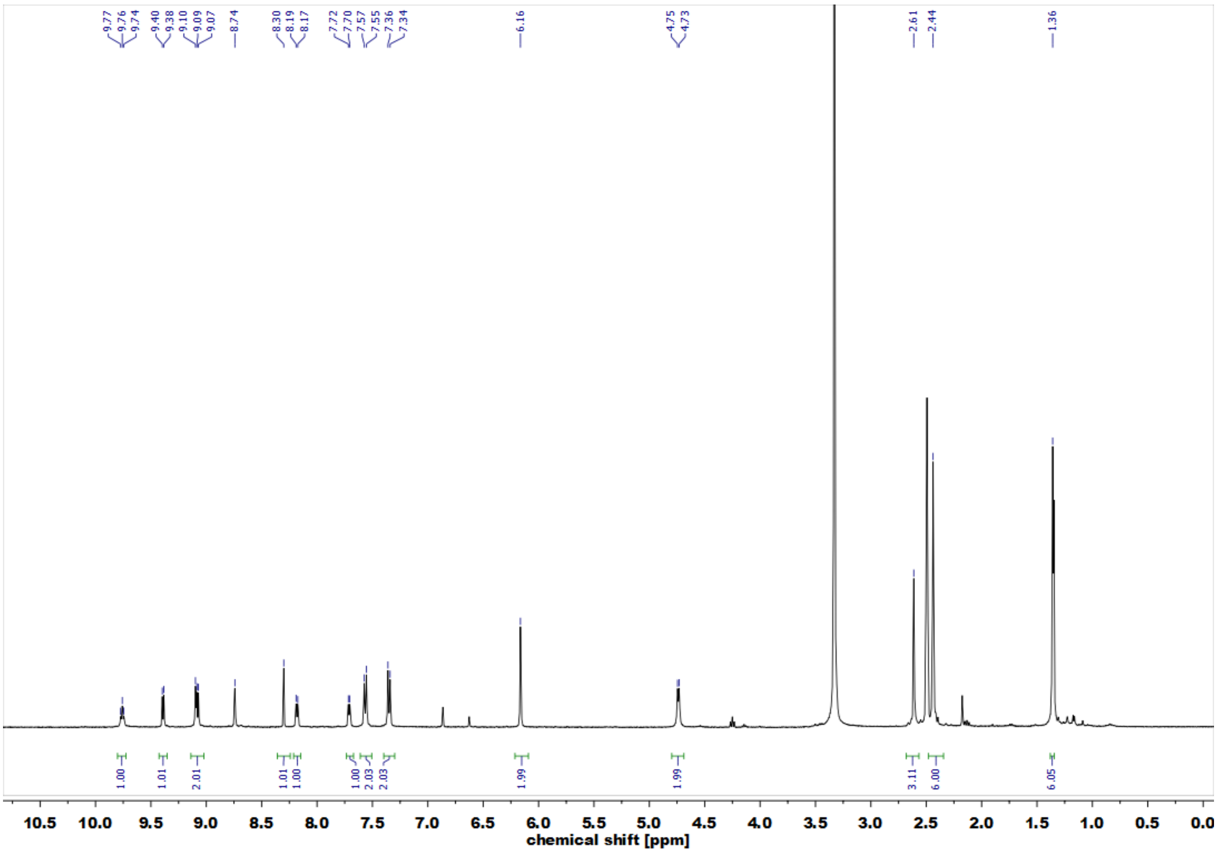

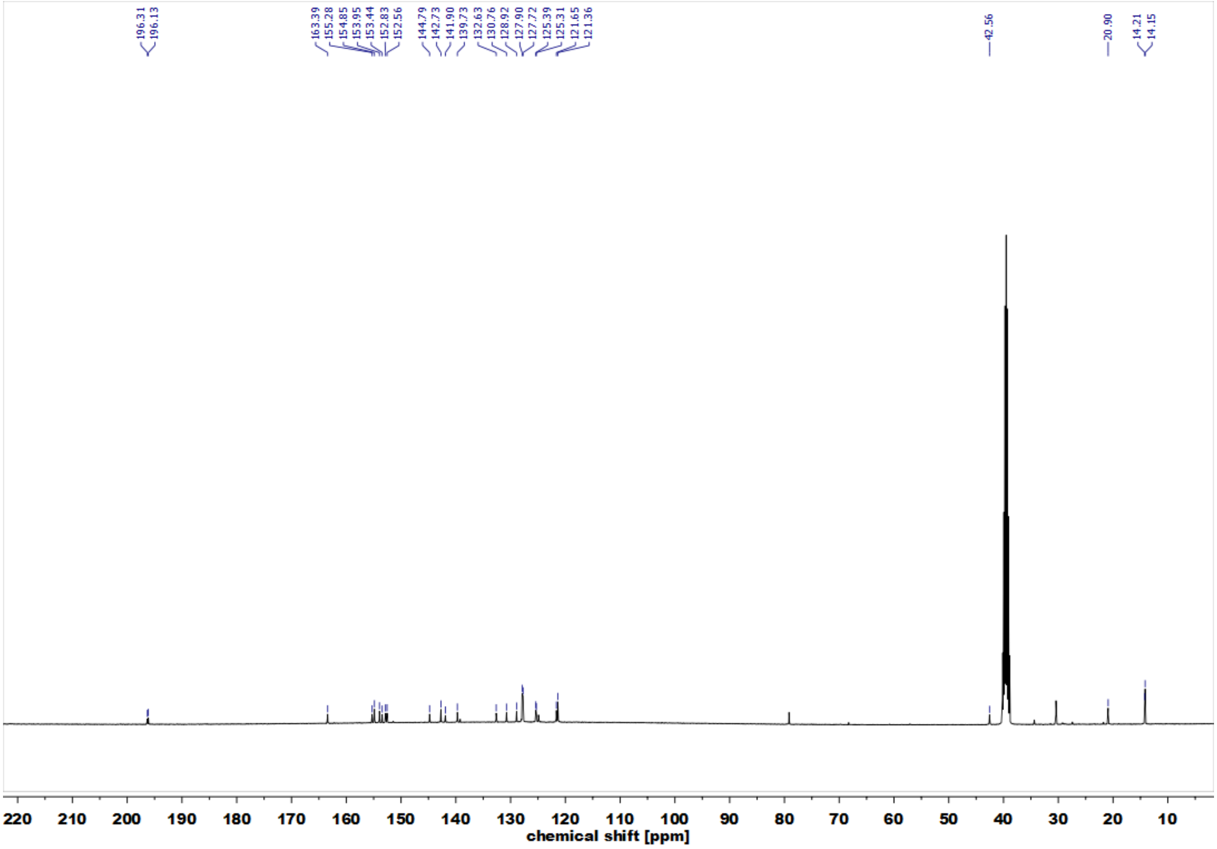

Figure S10. ^{1}H (top) and ^{13}C NMR (bottom) spectra of complex **10** measured in DMSO-d_6 at 25 °C.

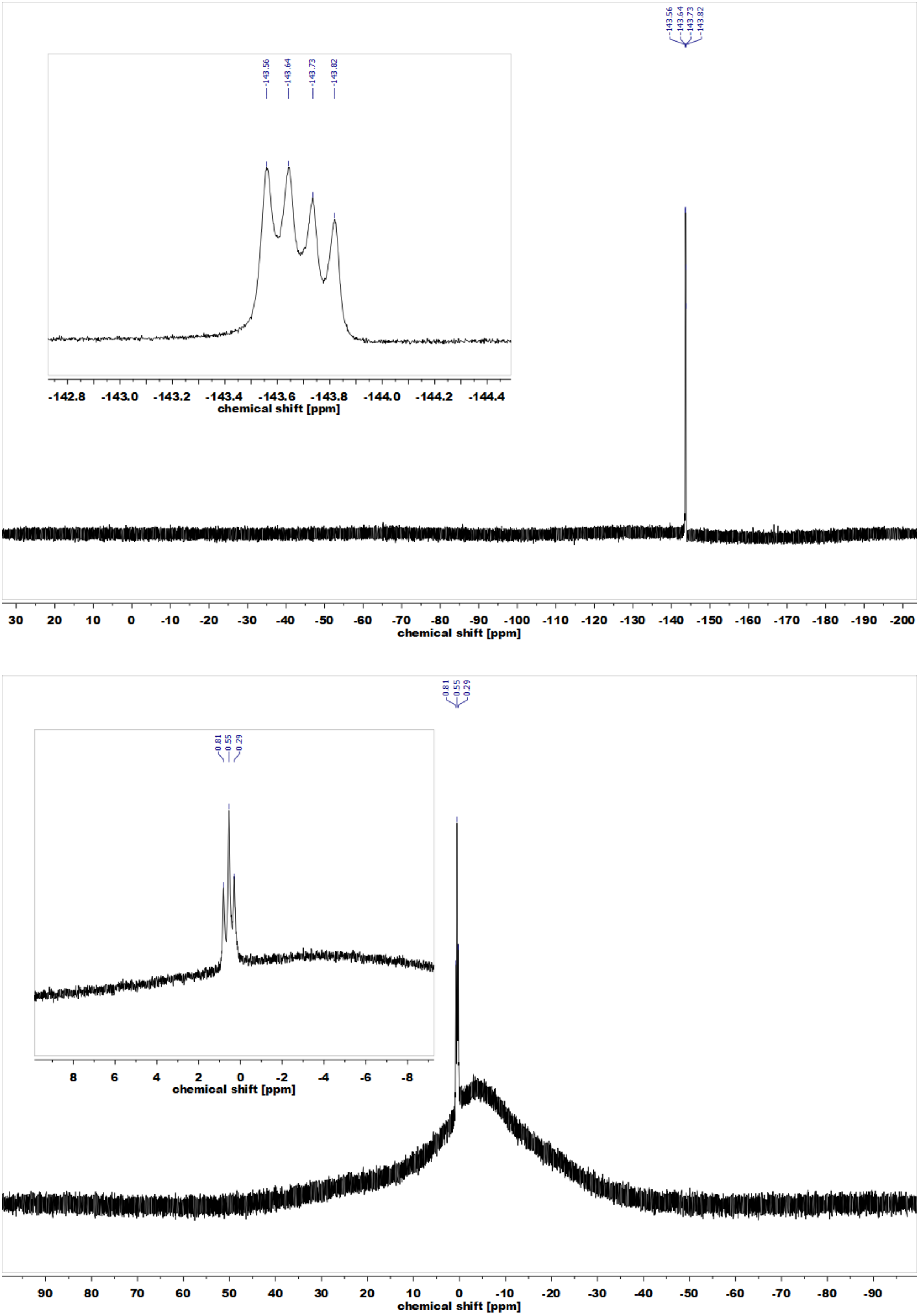

Figure S11. ^{19}F (top) and ^{11}B (bottom) spectra of complex **10** measured in DMSO-d_6 at 25 °C.

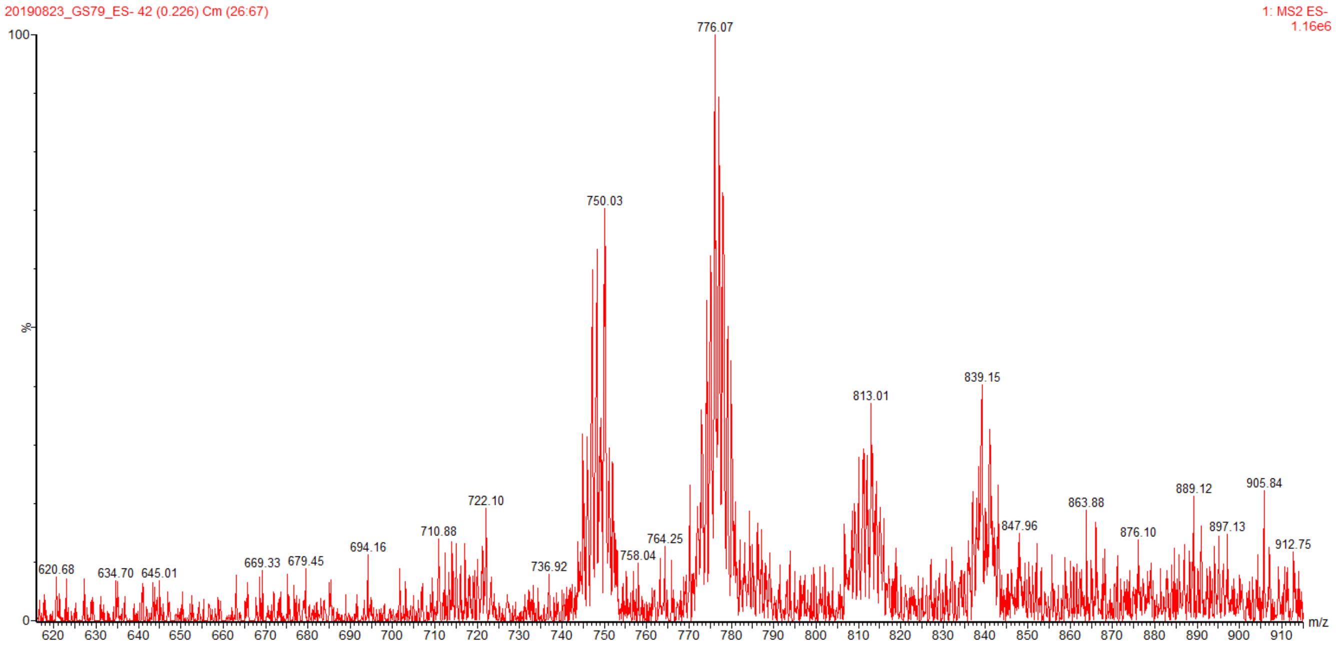

Figure S12. ESI (negative mode) mass spectrum of complex **10** in CH_3CN.

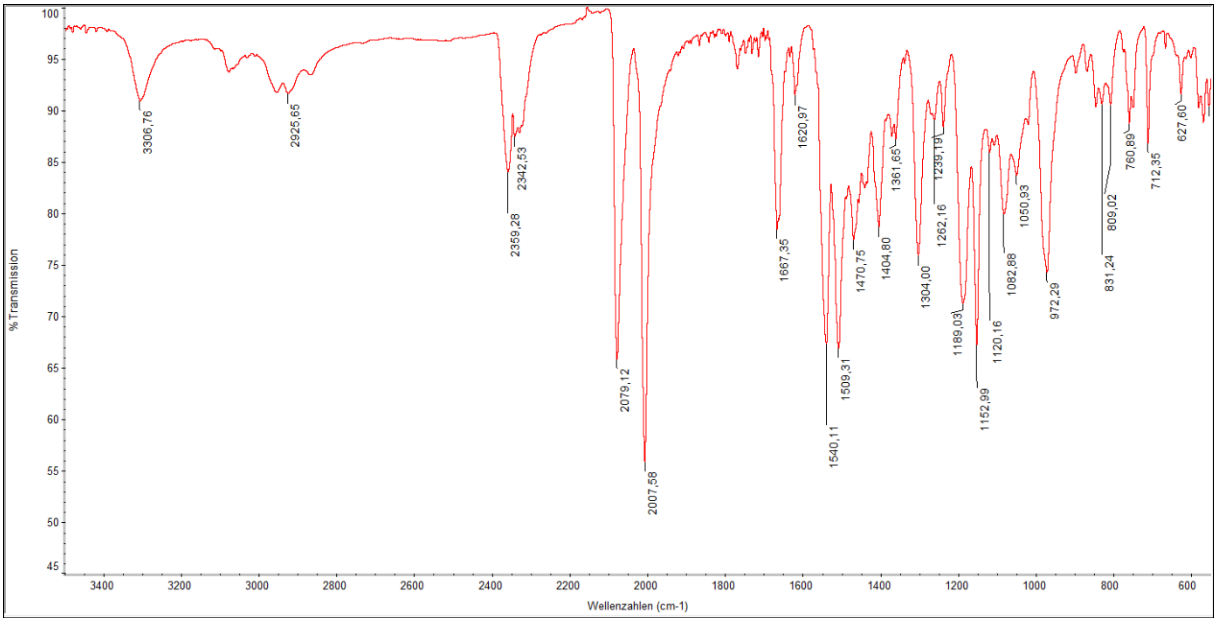

Figure S13. FT-IR spectrum of complex **10**.

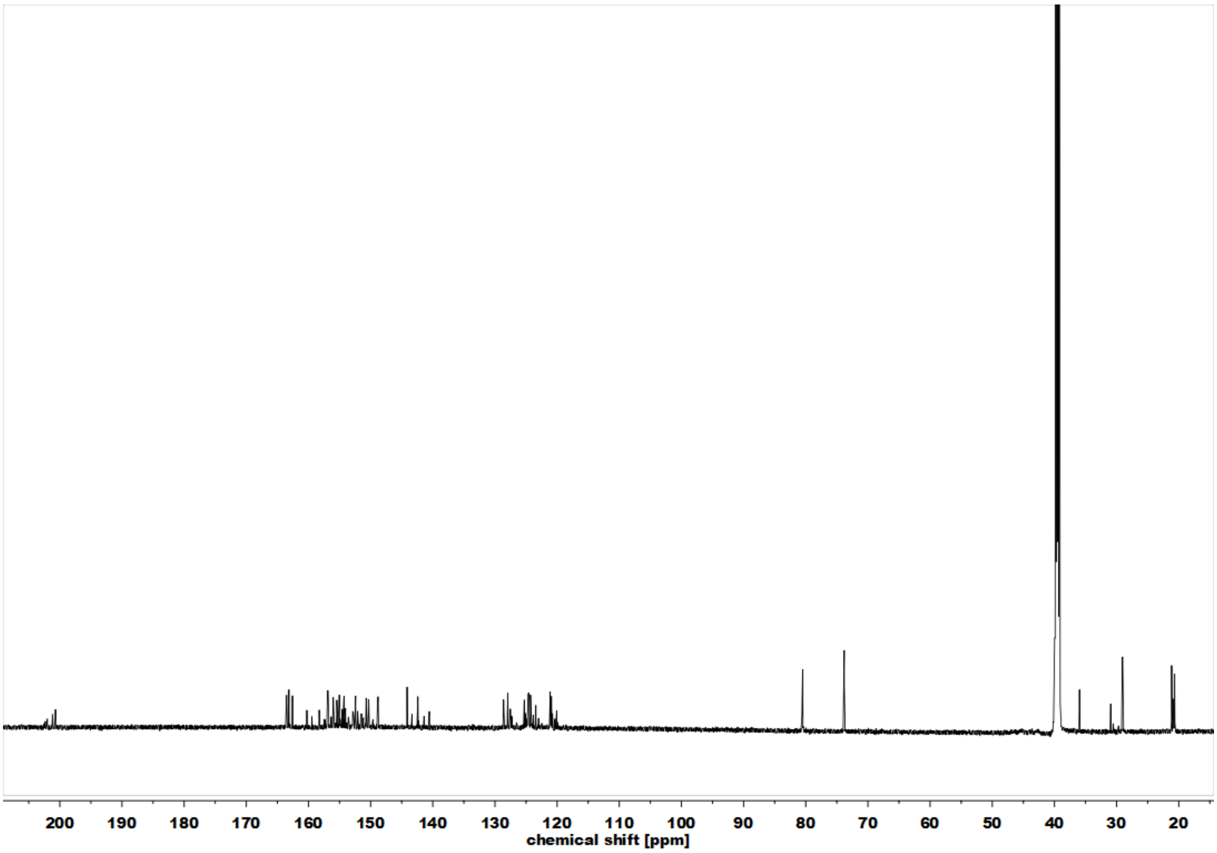

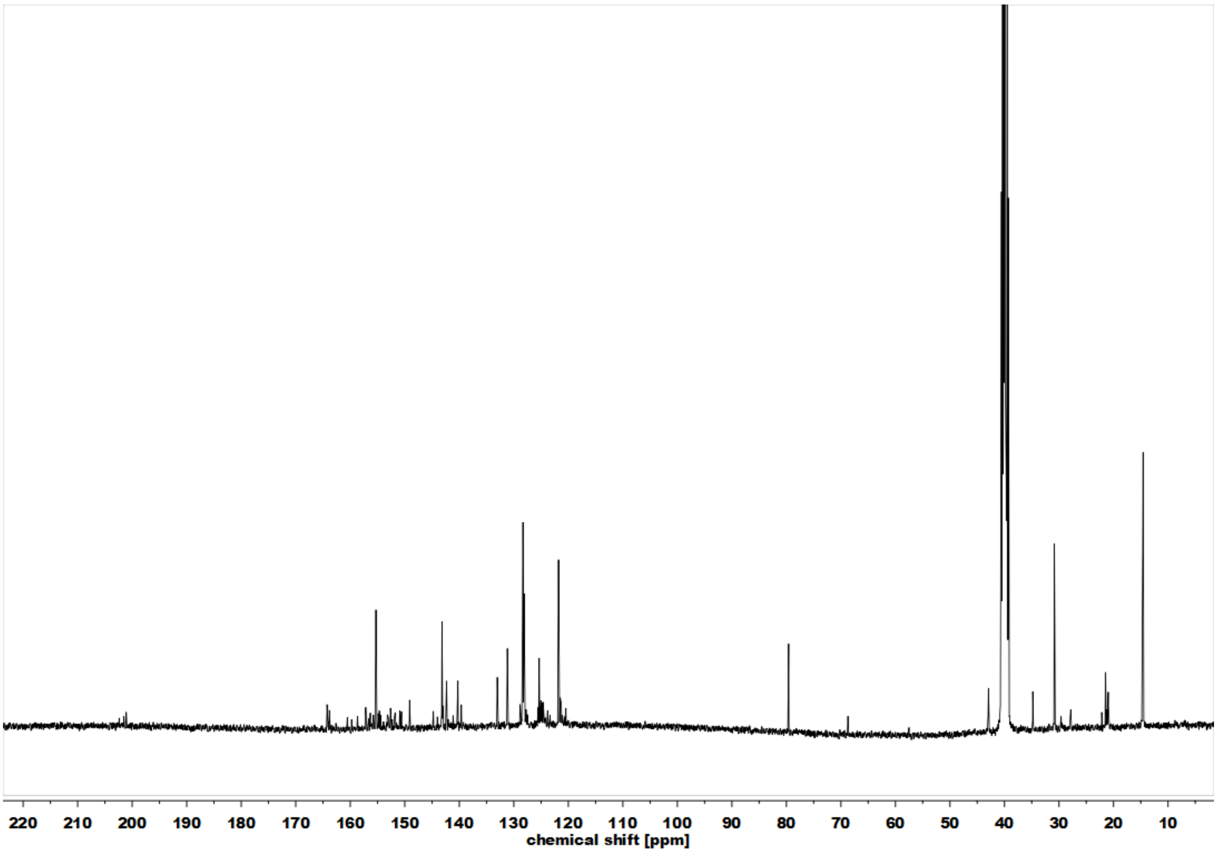

Figure S14. ^{13}C NMR spectra of complexes **5** (top) and **10** (bottom) measured in DMSO-d$_6$ at 25 °C after 4 h of exposure to 350 nm of radiation (E_v ~ 6 mW/cm^2).

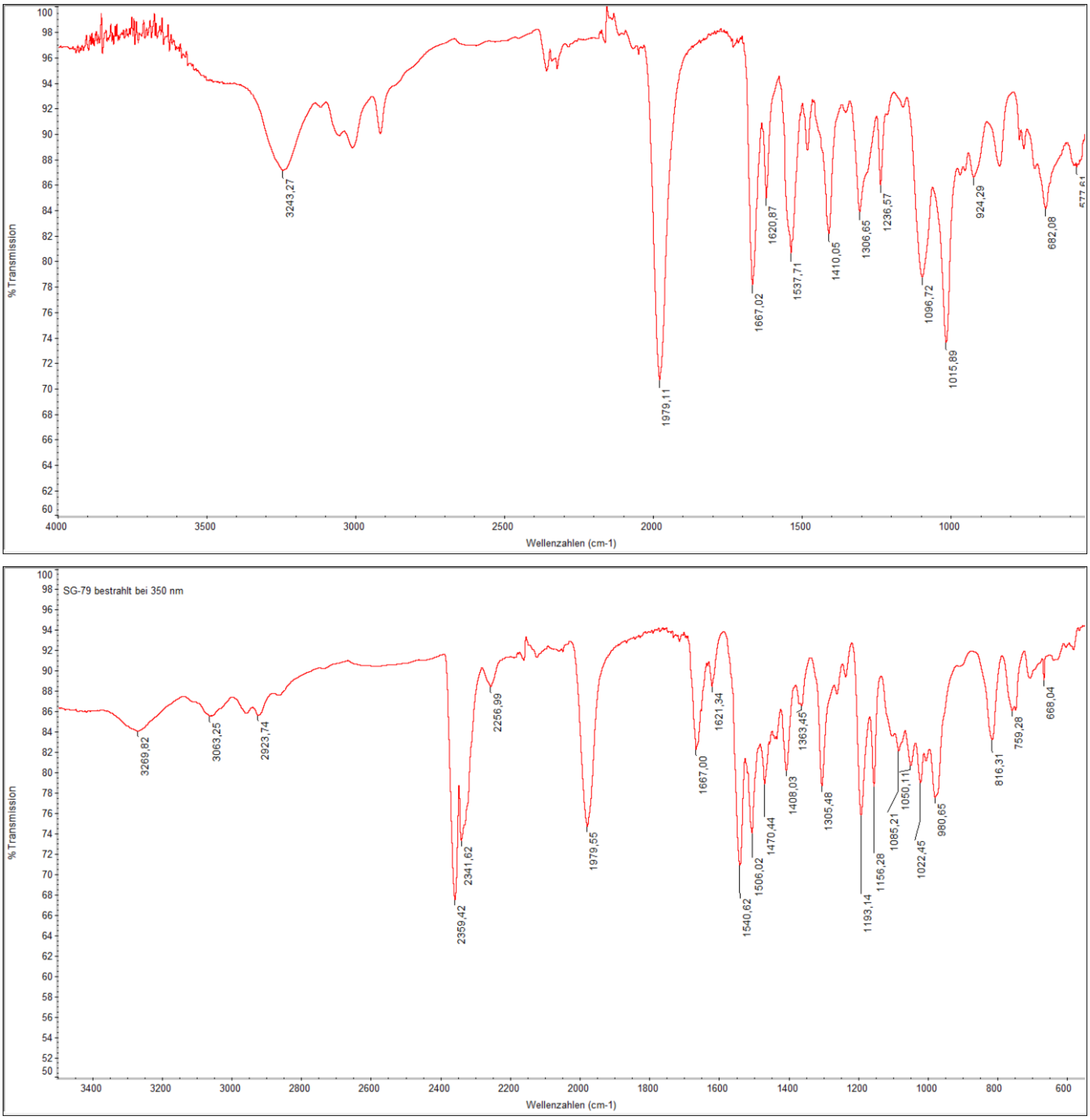

Figure S15. FT-IR spectra of complexes **5** (top) and **10** (bottom) after 4 h of exposure to 350 nm of radiation (E_v = 6 mW/cm^2).

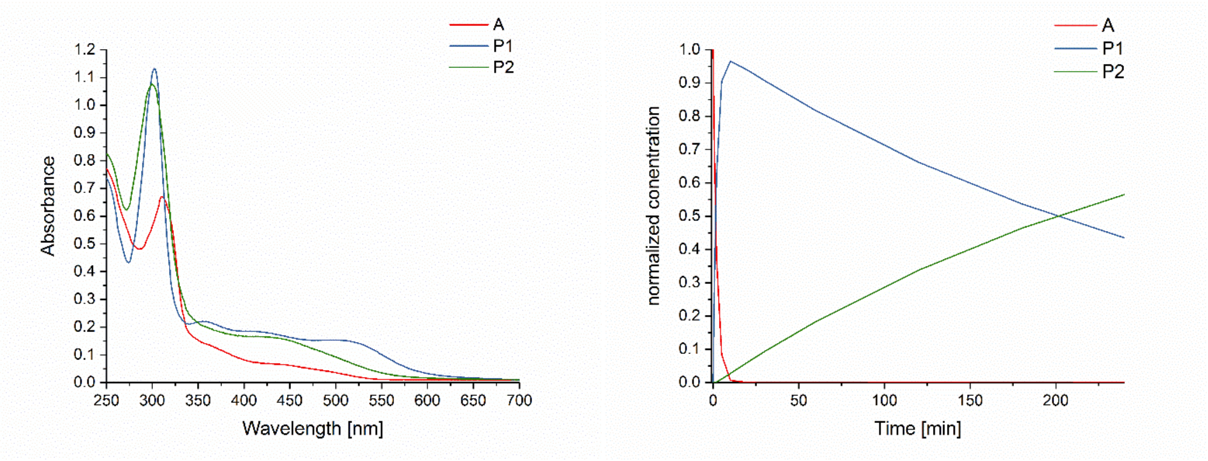

Figure S16. UV/Vis absorption spectra in water containing 0.8% (v/v) DMSO and concentration profiles for complex **5** (A) and the associated photoproducts (P1 and P2), derived by fitting the experimental data using MCR-ALS analysis.

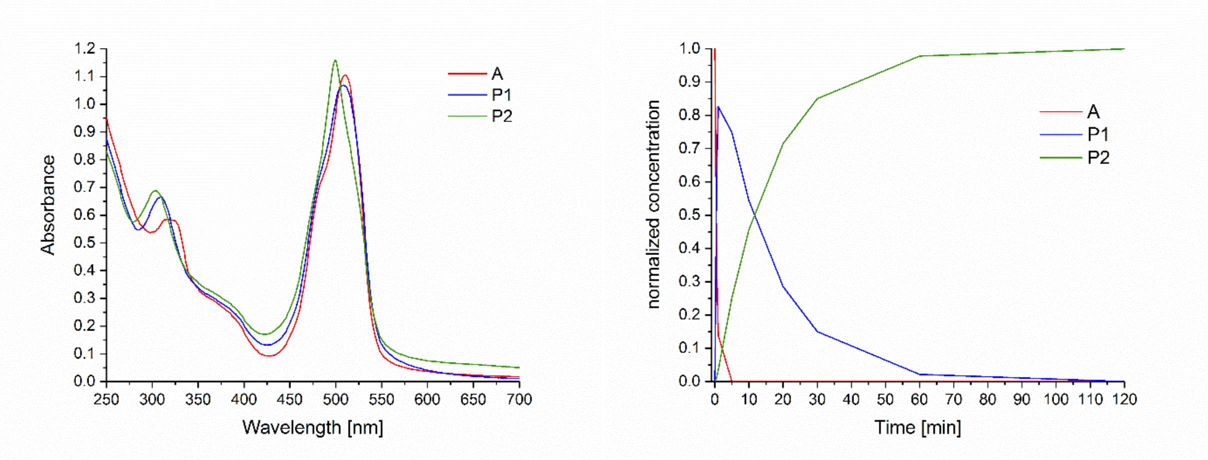

Figure S17. UV/Vis absorption spectra (left) in water containing 0.8% (v/v) DMSO and concentration profiles (right) for complex **10** (A) and the associated photoproducts (P1 and P2), derived by fitting the experimental data using MCR-ALS analysis.

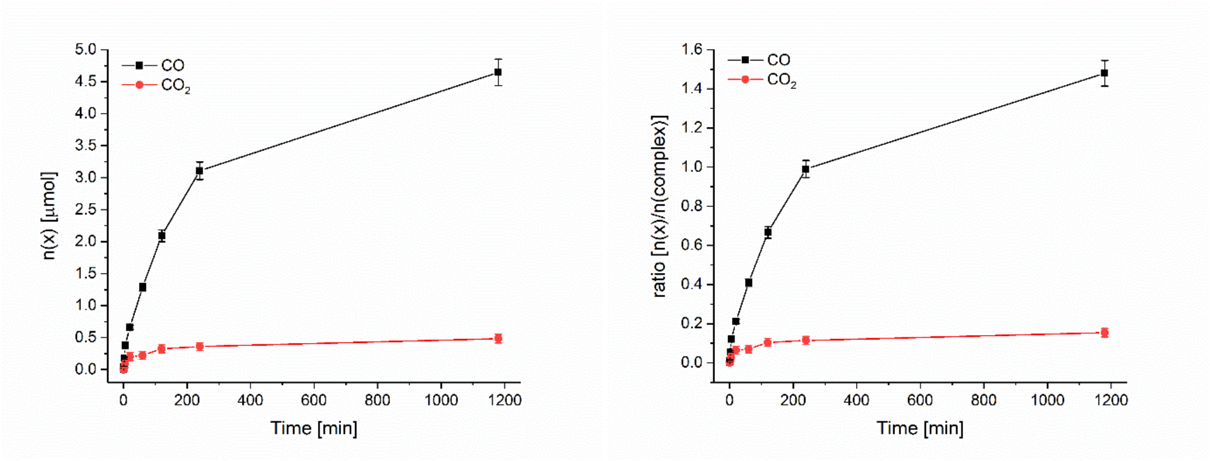

Figure S18. Amount of CO and CO_2 released from complex **5** (0.314 mM in DMSO/water (3:1, v/v)) upon exposure to 390 nm (0.35 µE/s) under N_2-atmosphere as function of time.

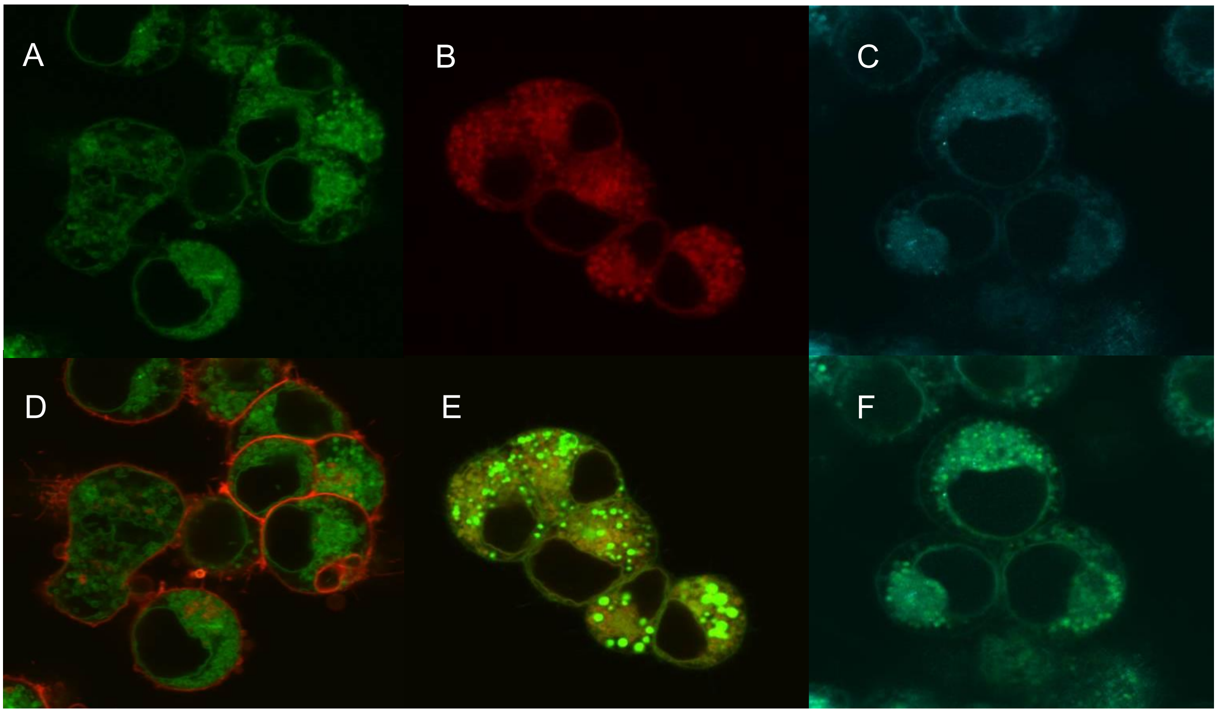

Figure S19. Cellular localisation of compound **10** (50 µM) incubated in HEK293 cells for 4 h in culture medium. Fluorescence image of **10** (A). Counter stains were then added for 15 to 30 min at 37 °C with 5% CO_2. Mitotracker^{TM} (0.25 µM) for mitochondrial staining (B), merged with compound **10** in (E), ER-Tracker Blue-White DPX (0.5 µM) for endoplasmic reticulum staining (C), merged with **10** in (F), Cellmask^{TM} Deep Red for plasma membrane staining, merged with **10** in (D).

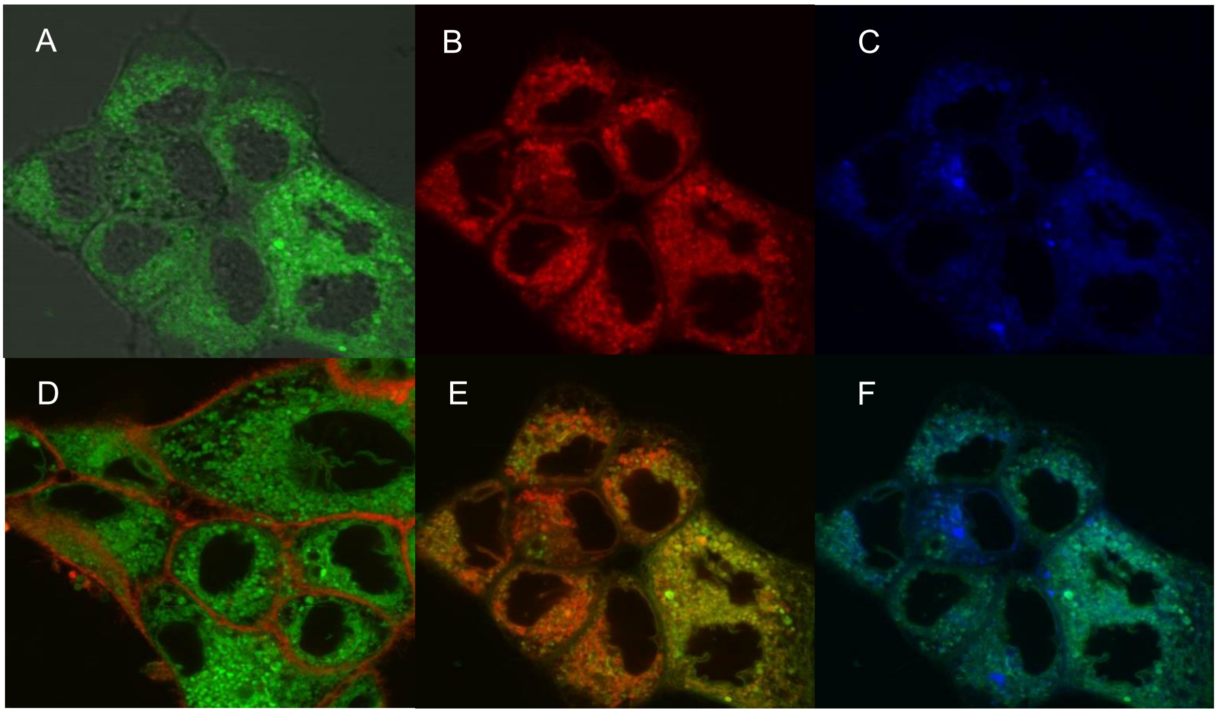

Figure S20. Cellular localisation of compound **10** (50 µM) incubated in A431 cells for 4 h in culture medium. Fluorescence image of **10** (A). Counter stains were then added for 15 to 30 min at 37 °C with 5% CO_2. Mitotracker^{TM} (0.25 µM) for mitochondrial staining (B), merged with compound **10** in (E), ER-Tracker Blue-White DPX (0.5 µM) for endoplasmic reticulum staining (C), merged with **10** in (F), Cellmask^{TM} Deep Red for plasma membrane staining (merged with **10** in (D).

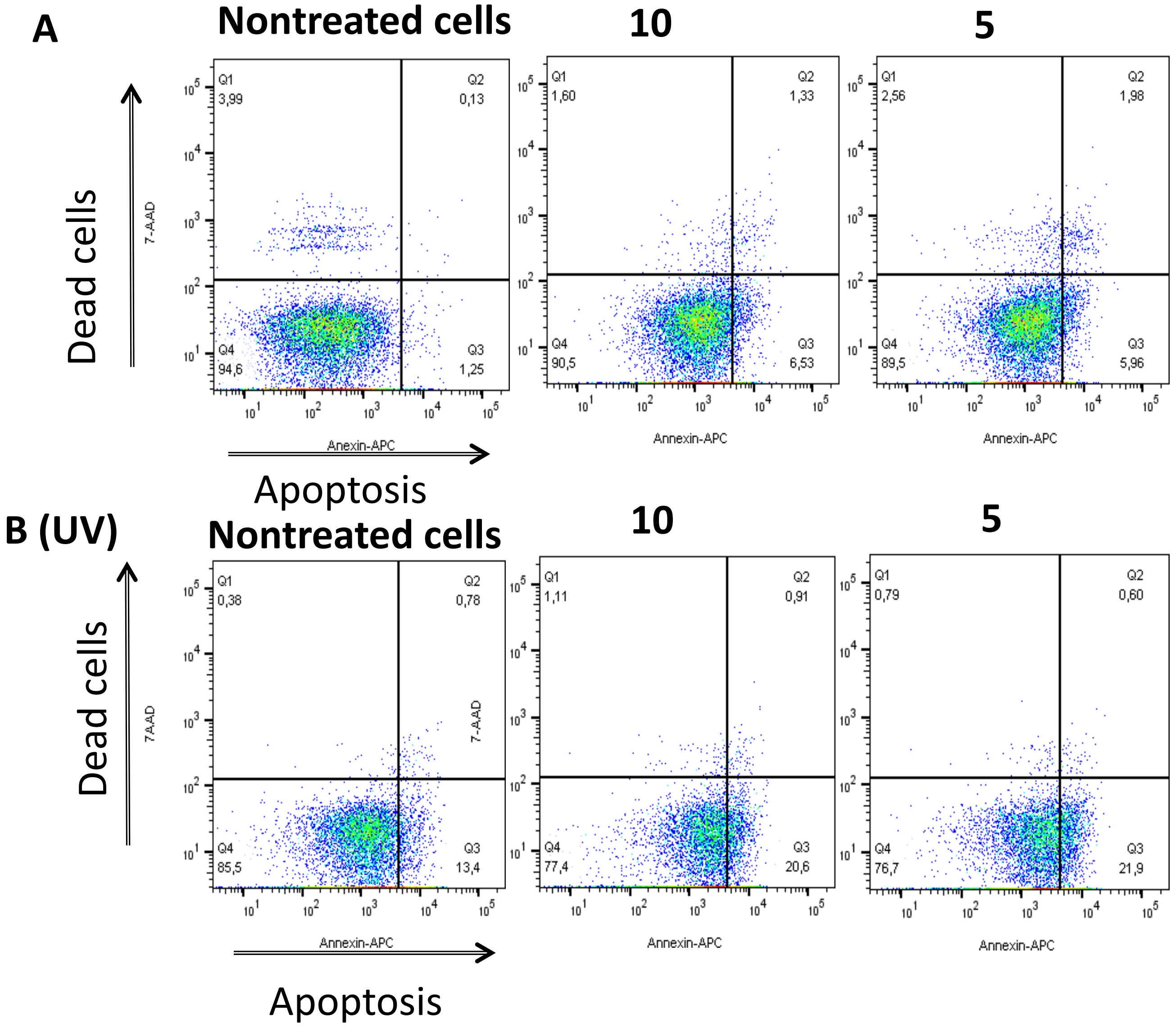

Figure S21. Flow cytometry analysis to determine the fractions of dead and apoptotic A431 cells. Cells were incubated with **5** and **10** for 30 min, then irradiated for 10 min and analysed after 4 h of incubation with fresh medium. Cells were labelled with 7-AAD to detect dead and AnexinV-APC to detect apoptotic cells.

Cells before (A) and after irradiation with UV light for 10 min (B); control (left); **5** (10 µM, right) and **10** (10 µM, middle). The dot plot graphs are divided into four compartments: Q1 – dead cells, Q2 – late apoptosis, Q3 – early apoptosis, Q4 – live cells.
